# Diretriz Brasileira Baseada em Evidências de 2025 para o Manejo da Obesidade e Prevenção de Doenças Cardiovasculares e Complicações Associadas à Obesidade: Uma Declaração de Posicionamento de Cinco Sociedades Médicas

**DOI:** 10.36660/abc.20250621

**Published:** 2025-10-13

**Authors:** José Francisco Kerr Saraiva, Cynthia M. Valerio, Fabiana H. Rached, Simone Van de Sande-Lee, Viviane Z. Rocha Giraldez, Fernando Valente, Luciano F. Drager, Bruno Halpern, Wellington S. da Silva, Fabio R. Trujilho, Neuton Dornellas, Ruy Lyra da Silva, João Eduardo N. Salles, Marcelo H. V. Assad, Marcio C. Mancini, Paulo A. C. Miranda, Rodrigo O. Moreira, Rodrigo N. Lamounier, Sergio E. Kaiser, Marcello C. Bertoluci

**Affiliations:** 1 Pontifícia Universidade Católica de Campinas Campinas SP Brasil Pontifícia Universidade Católica de Campinas, Campinas, SP – Brasil; 2 Instituto Estadual de Diabetes e Endocrinologia Luiz Capriglione Rio de Janeiro RJ Brasil Instituto Estadual de Diabetes e Endocrinologia Luiz Capriglione (IEDE-RJ), Rio de Janeiro, RJ – Brasil; 3 Hospital das Clínicas da Faculdade de Medicina da Universidade de São Paulo Instituto do Coração São Paulo SP Brasil Instituto do Coração (Incor) do Hospital das Clínicas da Faculdade de Medicina da Universidade de São Paulo (HCFMUSP), São Paulo, SP – Brasil; 4 Universidade Federal de Santa Catarina Florianópolis SC Brasil Universidade Federal de Santa Catarina (UFSC), Florianópolis, SC – Brasil; 5 Faculdade de Medicina do ABC São Paulo SP Brasil Faculdade de Medicina do ABC, São Paulo, SP – Brasil; 6 Centro de Controle de Peso do Hospital 9 de Julho São Paulo SP Brasil Centro de Controle de Peso do Hospital 9 de Julho, São Paulo, SP – Brasil; 7 Universidade Federal do Maranhão São Luís MA Brasil Universidade Federal do Maranhão (UFMA), São Luís, MA – Brasil; 8 Instituição Centro de Diabetes e Endocrinologia da Bahia Salvador BA Brasil Instituição Centro de Diabetes e Endocrinologia da Bahia (CEDEBA), Salvador, BA – Brasil; 9 Hospital da Obesidade Salvador BA Brasil Hospital da Obesidade, Salvador, BA – Brasil; 10 Corpo de Bombeiros Militar do Distrito Federal DF Brasil Corpo de Bombeiros Militar do Distrito Federal, DF – Brasil; 11 Universidade Federal de Pernambuco Recife PE Brasil Universidade Federal de Pernambuco (UFPE), Recife, PE – Brasil; 12 Faculdade de Ciências Médicas da Santa Casa de São Paulo São Paulo SP Brasil Faculdade de Ciências Médicas da Santa Casa de São Paulo, São Paulo, SP – Brasil; 13 Instituto Nacional de Cardiologia Rio de Janeiro RJ Brasil Instituto Nacional de Cardiologia (INC), Rio de Janeiro, RJ – Brasil; 14 Faculdade de Medicina da Universidade de São Paulo Hospital das Clínicas São Paulo SP Brasil Hospital das Clínicas da Faculdade de Medicina da Universidade de São Paulo (USP), São Paulo, SP – Brasil; 15 Associação Brasileira para o Estudo da Obesidade e Síndrome Metabólica São Paulo SP Brasil Associação Brasileira para o Estudo da Obesidade e Síndrome Metabólica (ABESO), São Paulo, SP – Brasil; 16 Santa Casa de Belo Horizonte Belo Horizonte MG Brasil Santa Casa de Belo Horizonte, Belo Horizonte, MG – Brasil; 17 Rede Mater Dei de Saúde Belo Horizonte MG Brasil Rede Mater Dei de Saúde, Belo Horizonte, MG – Brasil; 18 Centro Universitário Presidente Antônio Carlos Juiz de Fora MG Brasil Centro Universitário Presidente Antônio Carlos (UNIPAC), Juiz de Fora, MG – Brasil; 19 Universidade Federal de Minas Gerais Belo Horizonte MG Brasil Universidade Federal de Minas Gerais (UFMG), Belo Horizonte, MG – Brasil; 20 Centro de Diabetes de Belo Horizonte Belo Horizonte MG Brasil Centro de Diabetes de Belo Horizonte (CDBH), Belo Horizonte, MG – Brasil; 21 Universidade estadual do Rio de Janeiro Rio de Janeiro RJ Brasil Universidade estadual do Rio de Janeiro (UERJ), Rio de Janeiro, RJ – Brasil; 22 Universidade Federal do Rio Grande do Sul Porto Alegre RS Brasil Universidade Federal do Rio Grande do Sul (UFRGS), Porto Alegre, RS – Brasil

**Table:** 

Diretriz Brasileira Baseada em Evidências de 2025 para o Manejo da Obesidade e Prevenção de Doenças Cardiovasculares e Complicações Associadas à Obesidade: Uma Declaração de Posicionamento de Cinco Sociedades Médicas	
O relatório abaixo lista as declarações de interesse conforme relatadas à SBC pelos especialistas durante o período de desenvolvimento deste posicionamento, 2024/2025.	
Especialista	Tipo de relacionamento com a indústria
Bruno Halpern	Declaração financeira A - Pagamento de qualquer espécie e desde que economicamente apreciáveis, feitos a (i) você, (ii) ao seu cônjuge/ companheiro ou a qualquer outro membro que resida com você, (iii) a qualquer pessoa jurídica em que qualquer destes seja controlador, sócio, acionista ou participante, de forma direta ou indireta, recebimento por palestras, aulas, atuação como proctor de treinamentos, remunerações, honorários pagos por participações em conselhos consultivos, de investigadores, ou outros comitês, etc. Provenientes da indústria farmacêutica, de órteses, próteses, equipamentos e implantes, brasileiras ou estrangeiras: - Novo Nordisk, Lilly, Boehringer Ingelheim, Astra Zeneca, Merck, Novartis.
Cynthia M. Valerio	Declaração financeira A - Pagamento de qualquer espécie e desde que economicamente apreciáveis, feitos a (i) você, (ii) ao seu cônjuge/ companheiro ou a qualquer outro membro que resida com você, (iii) a qualquer pessoa jurídica em que qualquer destes seja controlador, sócio, acionista ou participante, de forma direta ou indireta, recebimento por palestras, aulas, atuação como proctor de treinamentos, remunerações, honorários pagos por participações em conselhos consultivos, de investigadores, ou outros comitês, etc. Provenientes da indústria farmacêutica, de órteses, próteses, equipamentos e implantes, brasileiras ou estrangeiras: - Novo Nordisk: Diabetes e Obesidade; Boehringer Ingelheim: Diabetes; Eli Lilly: Diabetes e Obesidade; AstraZeneca: Diabetes; EMS: Diabetes e Obesidade. B - Financiamento de pesquisas sob sua responsabilidade direta/pessoal (direcionado ao departamento ou instituição) provenientes da indústria farmacêutica, de órteses, próteses, equipamentos e implantes, brasileiras ou estrangeiras: - Chiesi: metreleptina. Outros relacionamentos Financiamento de atividades de educação médica continuada, incluindo viagens, hospedagens e inscrições para congressos e cursos, provenientes da indústria farmacêutica, de órteses, próteses, equipamentos e implantes, brasileiras ou estrangeiras: - Chiesi: metreleptina.
Fabiana H. Rached	Declaração financeira A - Pagamento de qualquer espécie e desde que economicamente apreciáveis, feitos a (i) você, (ii) ao seu cônjuge/ companheiro ou a qualquer outro membro que resida com você, (iii) a qualquer pessoa jurídica em que qualquer destes seja controlador, sócio, acionista ou participante, de forma direta ou indireta, recebimento por palestras, aulas, atuação como proctor de treinamentos, remunerações, honorários pagos por participações em conselhos consultivos, de investigadores, ou outros comitês, etc. Provenientes da indústria farmacêutica, de órteses, próteses, equipamentos e implantes, brasileiras ou estrangeiras: - Novo Nordisk: semaglutida; Novartis: inclisirana; Daiichi Sankyo: ácido bempedoico. Outros relacionamentos Financiamento de atividades de educação médica continuada, incluindo viagens, hospedagens e inscrições para congressos e cursos, provenientes da indústria farmacêutica, de órteses, próteses, equipamentos e implantes, brasileiras ou estrangeiras: - Novo Nordisk: semaglutida; Novartis: inclisirana; Daiichi Sankyo: ácido bempedoico.
Fabio R. Trujilho	Declaração financeira A - Pagamento de qualquer espécie e desde que economicamente apreciáveis, feitos a (i) você, (ii) ao seu cônjuge/ companheiro ou a qualquer outro membro que resida com você, (iii) a qualquer pessoa jurídica em que qualquer destes seja controlador, sócio, acionista ou participante, de forma direta ou indireta, recebimento por palestras, aulas, atuação como proctor de treinamentos, remunerações, honorários pagos por participações em conselhos consultivos, de investigadores, ou outros comitês, etc. Provenientes da indústria farmacêutica, de órteses, próteses, equipamentos e implantes, brasileiras ou estrangeiras: - Aché, Amryt Pharma, Astra Zeneca, BracePharma, EMS, Eurofarma, Lilly, Merck, NovoNordisk, Scitech Medical, Takeda.
Fernando Valente	Declaração financeira A - Pagamento de qualquer espécie e desde que economicamente apreciáveis, feitos a (i) você, (ii) ao seu cônjuge/ companheiro ou a qualquer outro membro que resida com você, (iii) a qualquer pessoa jurídica em que qualquer destes seja controlador, sócio, acionista ou participante, de forma direta ou indireta, recebimento por palestras, aulas, atuação como proctor de treinamentos, remunerações, honorários pagos por participações em conselhos consultivos, de investigadores, ou outros comitês, etc. Provenientes da indústria farmacêutica, de órteses, próteses, equipamentos e implantes, brasileiras ou estrangeiras: - Novo Nordisk: Diabetes e Obesidade; Boehringer Ingelheim: Diabetes; Eli Lilly: Diabetes e Obesidade, Abbott: CGM, AstraZeneca: Diabetes; Servier: Diabetes; EMS: Diabetes e Obesidade; GSK: Vacinas. Outros relacionamentos Financiamento de atividades de educação médica continuada, incluindo viagens, hospedagens e inscrições para congressos e cursos, provenientes da indústria farmacêutica, de órteses, próteses, equipamentos e implantes, brasileiras ou estrangeiras: - AstraZeneca: Diabetes; NovoNordisk: Diabetes.
João Eduardo N. Salles	Declaração financeira A - Pagamento de qualquer espécie e desde que economicamente apreciáveis, feitos a (i) você, (ii) ao seu cônjuge/ companheiro ou a qualquer outro membro que resida com você, (iii) a qualquer pessoa jurídica em que qualquer destes seja controlador, sócio, acionista ou participante, de forma direta ou indireta, recebimento por palestras, aulas, atuação como proctor de treinamentos, remunerações, honorários pagos por participações em conselhos consultivos, de investigadores, ou outros comitês, etc. Provenientes da indústria farmacêutica, de órteses, próteses, equipamentos e implantes, brasileiras ou estrangeiras: - Astra Zeneca, Bayer, Boehringer-Ingelheim, Lilly, Merck serono Novo Nordisk.
José Francisco Kerr Saraiva	Declaração financeira C - Financiamento de pesquisa (pessoal), cujas receitas tenham sido provenientes da indústria farmacêutica, de órteses, próteses, equipamentos e implantes, brasileiras ou estrangeiras: - Bayer: finerinona; Novo Nordisk: semaglutida; AstraZeneca: ciclosilicato de Zirconio, dapagliflozina; Amgen: evolocumabe; Boehringer Ingelheimer: empagliflozina; Lilly: tirzepatida, viatris atorvastatina; Daichii Sankyo: ácido bempedoico/Edoxabana; Mantecorp: rosuvastatina. Outros relacionamentos Financiamento de atividades de educação médica continuada, incluindo viagens, hospedagens e inscrições para congressos e cursos, provenientes da indústria farmacêutica, de órteses, próteses, equipamentos e implantes, brasileiras ou estrangeiras: - Bayer: finerinona; Novo Nordisk: Semaglutida; AstraZeneca: ciclosilicato de Zirconio, dapagliflozina; Amgen: evolocumabe; Boehringer Ingelheimer: empagliflozina; Lilly: tirzepatida, viatris atorvastatina; Daichii Sankyo: ácido bempedoico/edoxabana.
Luciano F. Drager	Declaração financeira A - Pagamento de qualquer espécie e desde que economicamente apreciáveis, feitos a (i) você, (ii) ao seu cônjuge/ companheiro ou a qualquer outro membro que resida com você, (iii) a qualquer pessoa jurídica em que qualquer destes seja controlador, sócio, acionista ou participante, de forma direta ou indireta, recebimento por palestras, aulas, atuação como proctor de treinamentos, remunerações, honorários pagos por participações em conselhos consultivos, de investigadores, ou outros comitês, etc. Provenientes da indústria farmacêutica, de órteses, próteses, equipamentos e implantes, brasileiras ou estrangeiras: - Lilly, Novo Nordisk, ResMed. Outros relacionamentos Financiamento de atividades de educação médica continuada, incluindo viagens, hospedagens e inscrições para congressos e cursos, provenientes da indústria farmacêutica, de órteses, próteses, equipamentos e implantes, brasileiras ou estrangeiras: - Novo Nordisk.
Marcello C. Bertoluci	Declaração financeira A - Pagamento de qualquer espécie e desde que economicamente apreciáveis, feitos a (i) você, (ii) ao seu cônjuge/ companheiro ou a qualquer outro membro que resida com você, (iii) a qualquer pessoa jurídica em que qualquer destes seja controlador, sócio, acionista ou participante, de forma direta ou indireta, recebimento por palestras, aulas, atuação como proctor de treinamentos, remunerações, honorários pagos por participações em conselhos consultivos, de investigadores, ou outros comitês, etc. Provenientes da indústria farmacêutica, de órteses, próteses, equipamentos e implantes, brasileiras ou estrangeiras: - AstraZeneca, NovoNordisk, Lilly, Boehringer, Bayer, Servier, Brace Pharma, Roche, AMGEM.
Marcelo H. V. Assad	Declaração financeira A - Pagamento de qualquer espécie e desde que economicamente apreciáveis, feitos a (i) você, (ii) ao seu cônjuge/ companheiro ou a qualquer outro membro que resida com você, (iii) a qualquer pessoa jurídica em que qualquer destes seja controlador, sócio, acionista ou participante, de forma direta ou indireta, recebimento por palestras, aulas, atuação como proctor de treinamentos, remunerações, honorários pagos por participações em conselhos consultivos, de investigadores, ou outros comitês, etc. Provenientes da indústria farmacêutica, de órteses, próteses, equipamentos e implantes, brasileiras ou estrangeiras: - AstraZeneca: Forxiga; BAYER: Firialta; Biolab: Repath; Boerhringer Ingelheim: Glyxambi; Daiichy Sankyo: Benicar, Nustendi; EMS: Bramicar; GSK: Shingrix; Libbs: Stanglit; Lilly: Mounjaro; Novo Nordisk: Wegovy; Novartis: Sybrava; Pfizer: Prevenar 20; Viatris: Lipitor, Inspra. B - Financiamento de pesquisas sob sua responsabilidade direta/pessoal (direcionado ao departamento ou instituição) provenientes da indústria farmacêutica, de órteses, próteses, equipamentos e implantes, brasileiras ou estrangeiras: - AMGEN: Olpasirana. Outros relacionamentos Financiamento de atividades de educação médica continuada, incluindo viagens, hospedagens e inscrições para congressos e cursos, provenientes da indústria farmacêutica, de órteses, próteses, equipamentos e implantes, brasileiras ou estrangeiras: - Bayer: Firialta; Daiichi Sankyo: Benicar; Novo Nordisk: Wegovy.
Marcio C. Mancini	Declaração financeira A - Pagamento de qualquer espécie e desde que economicamente apreciáveis, feitos a (i) você, (ii) ao seu cônjuge/ companheiro ou a qualquer outro membro que resida com você, (iii) a qualquer pessoa jurídica em que qualquer destes seja controlador, sócio, acionista ou participante, de forma direta ou indireta, recebimento por palestras, aulas, atuação como proctor de treinamentos, remunerações, honorários pagos por participações em conselhos consultivos, de investigadores, ou outros comitês, etc. Provenientes da indústria farmacêutica, de órteses, próteses, equipamentos e implantes, brasileiras ou estrangeiras: - Merck: Contrave; Novo Nordisk: Wegovy; EMS: Olyra; Myralis: Mecobe. B - Financiamento de pesquisas sob sua responsabilidade direta/pessoal (direcionado ao departamento ou instituição) provenientes da indústria farmacêutica, de órteses, próteses, equipamentos e implantes, brasileiras ou estrangeiras: - Abbott: FreeStyle Libre. Outros relacionamentos Financiamento de atividades de educação médica continuada, incluindo viagens, hospedagens e inscrições para congressos e cursos, provenientes da indústria farmacêutica, de órteses, próteses, equipamentos e implantes, brasileiras ou estrangeiras: - Novo Nordisk: Wegovy.
Neuton Dornellas	Declaração financeira A - Pagamento de qualquer espécie e desde que economicamente apreciáveis, feitos a (i) você, (ii) ao seu cônjuge/ companheiro ou a qualquer outro membro que resida com você, (iii) a qualquer pessoa jurídica em que qualquer destes seja controlador, sócio, acionista ou participante, de forma direta ou indireta, recebimento por palestras, aulas, atuação como proctor de treinamentos, remunerações, honorários pagos por participações em conselhos consultivos, de investigadores, ou outros comitês, etc. Provenientes da indústria farmacêutica, de órteses, próteses, equipamentos e implantes, brasileiras ou estrangeiras: - Novo Nordisk: uma reunião (Advisory board) sobre estudo SOUL - Semaglutida oral. Outros relacionamentos Financiamento de atividades de educação médica continuada, incluindo viagens, hospedagens e inscrições para congressos e cursos, provenientes da indústria farmacêutica, de órteses, próteses, equipamentos e implantes, brasileiras ou estrangeiras: - Novo Nordisk: inscrição, passagem e hospedagem para congresso europeu de obesidade.
Paulo A. C. Miranda	Declaração financeira A - Pagamento de qualquer espécie e desde que economicamente apreciáveis, feitos a (i) você, (ii) ao seu cônjuge/ companheiro ou a qualquer outro membro que resida com você, (iii) a qualquer pessoa jurídica em que qualquer destes seja controlador, sócio, acionista ou participante, de forma direta ou indireta, recebimento por palestras, aulas, atuação como proctor de treinamentos, remunerações, honorários pagos por participações em conselhos consultivos, de investigadores, ou outros comitês, etc. Provenientes da indústria farmacêutica, de órteses, próteses, equipamentos e implantes, brasileiras ou estrangeiras: - NovoNordisk, Lilly, Recordat, Ache, Ipsen.
Rodrigo N. Lamounier	Declaração financeira A - Pagamento de qualquer espécie e desde que economicamente apreciáveis, feitos a (i) você, (ii) ao seu cônjuge/ companheiro ou a qualquer outro membro que resida com você, (iii) a qualquer pessoa jurídica em que qualquer destes seja controlador, sócio, acionista ou participante, de forma direta ou indireta, recebimento por palestras, aulas, atuação como proctor de treinamentos, remunerações, honorários pagos por participações em conselhos consultivos, de investigadores, ou outros comitês, etc. Provenientes da indústria farmacêutica, de órteses, próteses, equipamentos e implantes, brasileiras ou estrangeiras: - Abbot: FreeStyle Libre; Minimed: Sistema 780G; EMS: Olira; Lilly: Mounjaro. Outros relacionamentos Financiamento de atividades de educação médica continuada, incluindo viagens, hospedagens e inscrições para congressos e cursos, provenientes da indústria farmacêutica, de órteses, próteses, equipamentos e implantes, brasileiras ou estrangeiras: - Novo Nordisk: Ozempic, Wegovy.
Rodrigo O. Moreira	Declaração financeira A - Pagamento de qualquer espécie e desde que economicamente apreciáveis, feitos a (i) você, (ii) ao seu cônjuge/ companheiro ou a qualquer outro membro que resida com você, (iii) a qualquer pessoa jurídica em que qualquer destes seja controlador, sócio, acionista ou participante, de forma direta ou indireta, recebimento por palestras, aulas, atuação como proctor de treinamentos, remunerações, honorários pagos por participações em conselhos consultivos, de investigadores, ou outros comitês, etc. Provenientes da indústria farmacêutica, de órteses, próteses, equipamentos e implantes, brasileiras ou estrangeiras: - Bayer, Astra Zeneca, Novo Nordisk, Eli Lilly, Libbs, EMS, Eurofarma, Chiesi, Servier. B - Financiamento de pesquisas sob sua responsabilidade direta/pessoal (direcionado ao departamento ou instituição) provenientes da indústria farmacêutica, de órteses, próteses, equipamentos e implantes, brasileiras ou estrangeiras: - Servier, AMRYT Pharma. Outros relacionamentos Financiamento de atividades de educação médica continuada, incluindo viagens, hospedagens e inscrições para congressos e cursos, provenientes da indústria farmacêutica, de órteses, próteses, equipamentos e implantes, brasileiras ou estrangeiras: - Astra Zeneca, Novo Nordisk, Bayer, Servier.
Ruy Lyra da Silva Filho	Declaração financeira A - Pagamento de qualquer espécie e desde que economicamente apreciáveis, feitos a (i) você, (ii) ao seu cônjuge/ companheiro ou a qualquer outro membro que resida com você, (iii) a qualquer pessoa jurídica em que qualquer destes seja controlador, sócio, acionista ou participante, de forma direta ou indireta, recebimento por palestras, aulas, atuação como proctor de treinamentos, remunerações, honorários pagos por participações em conselhos consultivos, de investigadores, ou outros comitês, etc. Provenientes da indústria farmacêutica, de órteses, próteses, equipamentos e implantes, brasileiras ou estrangeiras: - Abbott, Biolab, Boheringer, Lilly, Merck Serono, Eurofarma, MSD, Roche, Bayer, Mantecorp. Outros relacionamentos Financiamento de atividades de educação médica continuada, incluindo viagens, hospedagens e inscrições para congressos e cursos, provenientes da indústria farmacêutica, de órteses, próteses, equipamentos e implantes, brasileiras ou estrangeiras: - Abbott, Mantecorp, Boheringer, Lilly, Biolab, Eurofarma, Novo Nordisk, Bayer.
Sergio E. Kaiser	Declaração financeira A - Pagamento de qualquer espécie e desde que economicamente apreciáveis, feitos a (i) você, (ii) ao seu cônjuge/ companheiro ou a qualquer outro membro que resida com você, (iii) a qualquer pessoa jurídica em que qualquer destes seja controlador, sócio, acionista ou participante, de forma direta ou indireta, recebimento por palestras, aulas, atuação como proctor de treinamentos, remunerações, honorários pagos por participações em conselhos consultivos, de investigadores, ou outros comitês, etc. Provenientes da indústria farmacêutica, de órteses, próteses, equipamentos e implantes, brasileiras ou estrangeiras: - Bayer: Firialta; Novo Nordisk: Ozempic e Wegovy; Daiichi Sankyo: Nustendi e Benicar; Astrazeneca: Forxiga e Selozok; Novartis: Sybrava; Libbs: Plenance, Naprix; Biolab: Repatha e Livalo. Outros relacionamentos Financiamento de atividades de educação médica continuada, incluindo viagens, hospedagens e inscrições para congressos e cursos, provenientes da indústria farmacêutica, de órteses, próteses, equipamentos e implantes, brasileiras ou estrangeiras: - Novo Nordisk: Wegovy; Libbs: Naprix; Astrazeneca: Selozok.
Simone Van de Sande-Lee	Outros relacionamentos Financiamento de atividades de educação médica continuada, incluindo viagens, hospedagens e inscrições para congressos e cursos, provenientes da indústria farmacêutica, de órteses, próteses, equipamentos e implantes, brasileiras ou estrangeiras: - Abbott: Diabetes; Lilly: Obesidade e Diabetes; Novo Nordisk: Obesidade.
Viviane Z. Rocha Giraldez	Declaração financeira A - Pagamento de qualquer espécie e desde que economicamente apreciáveis, feitos a (i) você, (ii) ao seu cônjuge/ companheiro ou a qualquer outro membro que resida com você, (iii) a qualquer pessoa jurídica em que qualquer destes seja controlador, sócio, acionista ou participante, de forma direta ou indireta, recebimento por palestras, aulas, atuação como proctor de treinamentos, remunerações, honorários pagos por participações em conselhos consultivos, de investigadores, ou outros comitês, etc. Provenientes da indústria farmacêutica, de órteses, próteses, equipamentos e implantes, brasileiras ou estrangeiras: - Abbott: Lipidil; Aché: Trezete; Biolab: Repatha; Daichii-Sankyo: Nustendi; Lilly: Mounjaro; Novartis: Sybrava; Novo-Nordisk: Wegovy; Ultragenyx: Evkeeza. Outros relacionamentos Financiamento de atividades de educação médica continuada, incluindo viagens, hospedagens e inscrições para congressos e cursos, provenientes da indústria farmacêutica, de órteses, próteses, equipamentos e implantes, brasileiras ou estrangeiras: - Novartis: Sybrava; Novo-Nordisk: Wegovy.
Wellington S. da Silva Junior	Declaração financeira A - Pagamento de qualquer espécie e desde que economicamente apreciáveis, feitos a (i) você, (ii) ao seu cônjuge/ companheiro ou a qualquer outro membro que resida com você, (iii) a qualquer pessoa jurídica em que qualquer destes seja controlador, sócio, acionista ou participante, de forma direta ou indireta, recebimento por palestras, aulas, atuação como proctor de treinamentos, remunerações, honorários pagos por participações em conselhos consultivos, de investigadores, ou outros comitês, etc. Provenientes da indústria farmacêutica, de órteses, próteses, equipamentos e implantes, brasileiras ou estrangeiras: - Abbott, AstraZeneca, Libbs, Lilly, Novartis e Novo Nordisk.

## Sumário

**Introdução** 8
**Metodologia** 8
**Parte 1 – Definição do Risco Cardiovascular** 10
**1.1. Rastreamento para IC em Pessoas com Obesidade ou Sobrepeso** 10
**1.2. Rastreamento para IC em Pessoas com Obesidade ou Sobrepeso** 10
**Parte 2 – Metas de Perda de Peso** 13
**2.1. Meta de Perda de Peso para Redução de Fatores de Risco** 13
**2.2. Meta de Perda de Peso para Redução de Eventos Cardiovasculares** 13
**2.3. Objetivos de Redução Ponderal para Complicações Associadas à Fibrilação Atrial** 14
**Parte 3 – Manejo da Obesidade** 14
**3.1. Modificação do Estilo de Vida** 14
**3.2. Manejo da Farmacoterapia para Redução de Eventos e Fatores de Risco** 15
**3.2.1. Obesidade e Risco de DCVA Moderado ou Alto** 15
**3.2.2. Obesidade e Risco de DCVA Estabelecido** 16
**3.2.3. Obesidade e Diabetes Tipo 2** 18
**3.2.4. Obesidade, Diabetes Tipo 2 e Doença Renal Crônica** 18
**3.2.5. Obesidade e Apneia Obstrutiva do Sono** 18
**3.2.6. Obesidade e Insuficiência Cardíaca** 19
**3.2.7. Obesidade em Indivíduos com Alto Risco de Insuficiência Cardíaca** 11
**3.3. Cirurgia Bariátrica** 12
**3.3.1. Obesidade Estágio 2 e Risco de DCVA Moderado/Alto ou Alto Risco de IC** 12
**3.3.2. Obesidade Estágio 2 e Insuficiência Cardíaca** 13
**Conclusão** 24
**Suplemento 1** 24
**Referências** 25

## Introdução

A prevalência global da obesidade mais do que dobrou nas últimas quatro décadas, e hoje afeta mais de um bilhão de pessoas em todo o mundo. Reconhecida como uma condição ligada a muitas doenças crônicas, a obesidade impacta diretamente na qualidade de vida e na redução da expectativa de vida.^1^

Em 2021, a prevalência global da doença cardiovascular (DCV) atingiu 612 milhões de pessoas, representando 26,8% de todas as mortes mundiais, com um crescimento de 0,88% entre 1990 e 2021. Notavelmente, 79,5% de todos os anos de vida ajustados para incapacidades foram atribuídos a 11 fatores de risco, sendo o índice de massa corporal (IMC) o que apresentou a maior associação.^2^ Não obstante, estudos de prevalência mostram que dois terços das mortes relacionadas à obesidade são atribuíveis à DCV.^2 , 3^ Particularmente no Brasil, dados de 2025 sugerem que 68% dos adultos possuem IMC ≥ 25 kg/m², e que 31% dessa população esteja vivendo com a obesidade. Em 2021, houve 60.913 mortes prematuras associadas ao IMC elevado no país.^1^

A obesidade também é um dos principais determinantes da DCV. Estudos epidemiológicos prospectivos demonstram que a obesidade está associada a um risco elevado de eventos de doença arterial coronariana (DAC) e de mortalidade cardiovascular (CV).^4^ Sua influência ocorre de forma indireta, através do aumento de fatores de risco CV tradicionais como diabetes tipo 2 (DM2), dislipidemia (DLP) e hipertensão arterial sistêmica (HAS), mas também por efeito direto do estado inflamatório induzido pela adiposidade na estrutura e função cardíacas.^5 , 6^

Diversas evidências epidemiológicas ligam a obesidade à DCV através do IMC. Uma metanálise envolvendo mais de 300.000 adultos evidenciou que as faixas de sobrepeso e obesidade definidas pelo IMC se associam a um risco elevado de DAC e de mortalidade CV. Estudos observacionais e de randomização mendeliana também demonstram forte associação direta entre o aumento do IMC e a incidência e mortalidade por insuficiência cardíaca (IC).^7^

Adicionalmente, as evidências demonstram que a obesidade abdominal está mais diretamente relacionada ao aumento do risco de doenças cerebrovasculares, coronarianas, e mortalidade CV.^5^ Metanálises de grandes estudos de coorte evidenciam que a obesidade abdominal, medida pela circunferência da cintura, é forte preditora independente para morbidade e mortalidade em qualquer categoria de IMC.^5 , 6^ Mesmo indivíduos com IMC abaixo de 30 kg/m^2^ podem apresentar risco cardiometabólico alto relacionado à escassez de gordura subcutânea gluteofemoral e ao acúmulo de gordura visceral, especialmente quando associado a outros fatores de risco.^8^ Desta forma, alternativas ao IMC como a circunferência da cintura, a relação cintura/quadril e a relação cintura-altura são recomendadas para evidenciar o possível aumento de gordura visceral.^9 - 11^

Historicamente, a abordagem terapêutica para a obesidade tem se centrado em intervenções relacionadas ao estilo de vida e opções farmacológicas com eficácia limitada. Contudo, a introdução da nova geração de medicações antiobesidade possibilitou a obtenção de perdas de peso mais significativas e sustentáveis. Ademais, com a complexidade crescente das opções terapêuticas disponíveis e os benefícios observados em desfechos ligados à síndrome cardiorrenal metabólica; tornou-se evidente a necessidade de novas ferramentas de estratificação que orientem a seleção do tratamento para cada situação clínica.^12 , 13^

Nesse sentido, esta diretriz visa estruturar o tratamento da obesidade em relação à prevenção da DCV, levando em consideração o risco CV e o estágio da obesidade. Este documento apresenta recomendações fundamentadas nas melhores evidências disponíveis, com o objetivo de auxiliar os profissionais de saúde na personalização da estratégia terapêutica mais adequada para as pessoas que convivem com obesidade.

## Metodologia

A diretriz seguiu o método Delphi^14^ para estabelecer recomendações a partir da coleta de opiniões de especialistas em rodadas sucessivas, na qual cada participante responde anonimamente e tem a oportunidade de reavaliar suas respostas à luz do *feedback* dos outros participantes.

Inicialmente, formou-se um grupo de 20 especialistas representantes das 5 sociedades (ABESO, SBD, SBEM, SBC e ABS). Destes, 5 formaram o grupo de trabalho (comitê gestor), que elaborou a estrutura da diretriz baseada em 25 recomendações, elaboradas com as melhores evidências disponíveis.

Cada recomendação foi desenhada para abordar uma situação clínica específica, e recebeu um grau de recomendação que foi dado após votação do grande grupo. Foram realizadas 3 rodadas de votação por meio de uma ferramenta online, cujo resultado foi analisado estatisticamente pelo comitê gestor. Após a primeira rodada de sugestões, o texto base foi revisado e reescrito. Foram realizadas então a segunda e terceira rodadas para ajustes do texto, que foi subsequentemente ajustado para definição final dos graus de recomendação. Em seguida, a revisão da literatura foi atualizada e estruturada conforme o sumário de evidências que segue cada recomendação. Finalmente, o texto foi redigido para publicação.

O grau de recomendação e o nível de evidência de cada recomendação foram estabelecidos conforme as tabelas a seguir ( Tabelas 1 e 2 ):

**Figure:**
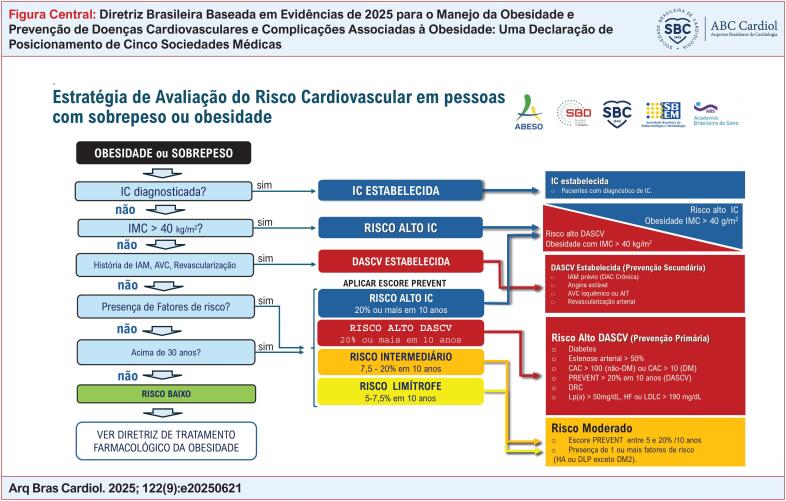


**Tabela 1:** Grau de recomendação

Termo	Definição	Classe de Recomendação.
É RECOMENDADO	90% ou mais concordam com a recomendação	I
DEVE SER CONSIDERADO	75-89% concordam com a recomendação	IIa
PODE SER CONSIDERADO	50-74% concordam com a recomendação	IIb
NÃO É RECOMENDADO	Menos de 50% concordam com a recomendação	III

**Tabela 2:** Nível de evidência

Nível de evidência	Definição	Cor
A	Dois ou mais ensaios clínicos randomizados e controlados (ECR), ou metanálise de ECR	
B	Metanálise de estudos observacionais, Meta Análise de subgrupos, Estudos observacionais longitudinais de grande porte, análises de subgrupos pré especificadas de grandes ECR,	
C	Estudos transversais, experimentos, estudos de casos e controles, série de casos, análises exploratórias, diretrizes de sociedades, opinião de experts	

## Parte 1 – Definição do Risco Cardiovascular

### 1.1. Avaliação do Risco Cardiovascular em Indivíduos com Sobrepeso ou Obesidade

**Table:** 

R1.	Grau de recomendação	Nível de evidência
**É RECOMENDADO** avaliar e categorizar o risco em relação à Doença Aterosclerótica Cardiovascular (DASCV) e à Insuficiência Cardíaca (IC) de todos os indivíduos adultos acima de 18 anos com sobrepeso ou obesidade, com o objetivo de orientar o tratamento da obesidade.	I	C

Sumário de evidências (R1):

- Em face ao surgimento de evidências de medicações com benefício CV, em termos de redução de risco DASCV e de risco de IC em pessoas com obesidade, considera-se que a escolha do tratamento farmacológico deva ser dirigida pela estratificação do risco CV, com o objetivo de otimizar a resposta ao tratamento. Essa recomendação é baseada em opinião de especialistas.

**Table:** 

R2.	Grau de recomendação	Nível de evidência
**É RECOMENDADO** o uso do escore de risco PREVENT para avaliação do risco CV em pessoas com sobrepeso ou obesidade com IMC menor que 40 kg/m^2^ e idade entre 30 e 79 anos, que estejam em prevenção primária, devendo o escore ser utilizado no modo que inclui o valor da HbA1c, utilizando o risco DASCV total e o risco IC em 10 anos.	I	C

Sumário de evidências (R2):

- O escore PREVENT ( *Predicting Risk of CVD events* ) foi preferido em relação ao modelo tradicional mais antigo, *Pooled Cohort Equations* (PCEs), em função da maior representatividade étnica, do número maior de populações incluídas e da maior acurácia. O escore PREVENT tem, entretanto, limitações quanto à abrangência de subtipos de DCV. O modelo PREVENT incorpora desfechos ampliados, incluindo IC e fatores de risco relacionados à obesidade, diabetes e doença renal. O desempenho prognóstico do modelo de risco demonstra boa discriminação e calibração tanto na população geral quanto entre os subgrupos demográficos e CV-renais-metabólicos.^15 , 16^
- Idealmente concebido para indivíduos com sobrepeso ou obesidade, o escore de risco PREVENT deve ser aplicado no modo de utilização que inclui a medida de HbA1c. Ambos os riscos DASCV total e o risco IC em 10 anos devem ser avaliados. O escore PREVENT possui limitações em relação à idade e aos níveis de IMC, devendo ter seu uso restrito a pacientes com idade entre 30-79 anos e IMC < 40 kg/m^2^.

**Table:** 

R3.	Grau de recomendação	Nível de evidência
**É RECOMENDADO** categorizar o risco CV dos indivíduos com obesidade e sobrepeso como: risco DASCV BAIXO, MODERADO ou ALTO e risco IC ALTO (de acordo com a Tabela 3 e Figura Central ).	I	C

**Table:** 

Nota importante 1: Quando reestratificar pessoas com RISCO MODERADO
Pessoas com risco moderado pelo escore PREVENT, com necessidade de reestratificar o risco DASCV por história familiar de DAC precoce, devem realizar o escore de cálcio coronário (CAC), medido por meio de tomografia computadorizada do tórax. As pessoas com CAC > 100 Ag (sem diabetes) e as com CAC > 10 Ag (com diabetes) devem ser estratificadas para RISCO ALTO. Pessoas com CAC = zero continuam no risco moderado se houver diabetes. Pacientes que apresentam diabetes tipo 2 há mais de 10 anos ou doença renal crônica (taxa de filtração glomerular estimada (TFG) < 45 ml/min/1,73m^2^ e/ou albuminúria, com relação albumina/creatinina (RAC) na urina > 30 mg/g) devem ser considerados de RISCO ALTO, independentemente do escore PREVENT.

### 1.2. Rastreamento para IC em Pessoas com Obesidade ou Sobrepeso

**Table:** 

R4.	Grau de recomendação	Nível de evidência
**DEVE SER CONSIDERADO** o rastreamento para IC através da dosagem dos peptídeos atriais (NT-proBNP ou BNP) ou por método de imagem em pessoas com RISCO IC ALTO (ver limites na nota importante 3).	IIa	C

**Tabela 3:** Categorização do Risco Cardiovascular em pessoas com sobrepeso ou obesidade

RISCO DASCV	DEFINIÇÃO
**BAIXO** < 5% em 10 anos	· Pessoas com sobrepeso ou obesidade com IMC < 40 kg/m² e idade menor que 30 anos, que não apresentam nenhum fator de risco CV (ver nota importante 1). · Indivíduos com sobrepeso ou obesidade com idade ≥30 anos, com risco CV total pelo escore PREVENT menor que 5% em 10 anos.
**MODERADO** 5 a 20% em 10 anos	· Pessoas com sobrepeso ou obesidade, com IMC < 40 kg/m² que nunca tiveram eventos CV, com 1 ou mais fatores de risco (ver suplemento 1). · Pessoas com sobrepeso ou obesidade, com IMC < 40 kg/m² em prevenção primária, com risco CV total pelo escore PREVENT entre 5% e < 20% em 10 anos.
**ALTO** > 20% em 10 anos	· Doença coronariana crônica confirmada, infarto agudo do miocárdio (IAM), acidente vascular cerebral (AVC) isquêmico ou acidente isquêmico transitório (AIT), doença arterial obstrutiva periférica (DAOP), revascularização em qualquer território arterial. Prevenção primária, com risco CV total pelo escore PREVENT maior ou igual a 20% em 10 anos. Diabetes tipo 2 há mais de 10 anos. Doença renal crônica 3b (ver Nota importante 1). Escore de cálcio coronário (CAC) > 100 Ag (sem diabetes) e CAC > 10 Ag (com diabetes), Lp (a) > 50 mg/dL ou Lp(a) > 125 nmol/L, HF ou LDL > 190 mg/dL.
**RISCO ALTO IC** > 20% em 10 anos	· IMC > 40kg/m^2^, mesmo assintomáticas. · Pessoas com obesidade, diabetes e hipertensão associados. · Apneia obstrutiva do sono (AOS) grave · Fibrilação Atrial. · Doença renal crônica grau 3b (ver Nota importante 1). · NTproBNP/BNP elevados. · Escore PREVENT para IC em 10 anos igual ou maior que 20%. · DASCV estabelecida. Sintomas sugestivos de IC.

**Observação:** Para fins de simplificação, a categoria de risco moderado engloba os riscos originais limítrofe (5-7.5%/20 anos) e intermediário (7,5 a < 20%/20 anos).

**Legendas:** DCV: Doença Cardiovascular; IC: Insuficiência Cardíaca; AVC: Acidente Vascular Cerebral isquêmico; REVASC: Cirurgia de Revascularização Arterial; DAC: Doença Arterial Coronariana; HA: Hipertensão Arterial; DM2: Diabetes tipo 2; DLP: Dislipidemia; DRC: Doença Renal Crônica. QV: Qualidade de vida; CAC: escore de cálcio coronário; HF: Hipercolesterolemia familiar; AOS: apneia obstrutiva do sono; Lp(a): Lipoproteína (a); LDL: lipoproteína de baixa densidade

Sumário de evidências (R4):

- A obesidade é um importante fator de risco para a IC com fração de ejeção preservada. Indivíduos com obesidade tendem a receber o diagnóstico tardiamente, com quadros já avançados de disfunção cardíaca, já que os sintomas muitas vezes são atribuídos à obesidade *per se* , especialmente em estágios mais avançados.^17^
- Para pessoas com obesidade e RISCO IC ALTO, o rastreio de diabetes tipo 2, hipertensão, fibrilação atrial, apneia obstrutiva do sono (AOS) e evidências objetivas de intolerância ao exercício podem identificar a necessidade de intervenções direcionadas para a IC. O tratamento oportuno da IC irá melhorar o seu prognóstico relacionado à qualidade de vida e à morbimortalidade.^18^
- A associação entre o índice de apneia-hipopneia (IAH) e mortalidade cardiovascular é positiva, mas de magnitude moderada. Uma meta-análise encontrou HR de 2,07 (IC 95%: 1,48-2,91) para mortalidade CV na AOS grave (IAH ≥ 30 eventos/hora) versus controles, enquanto outro estudo encontrou um risco relativo de 1,79 (IC 95%: 1,47-2,18) para eventos cardiovasculares neste estágio.^19^ Análises de dose-resposta mostram que cada aumento de 10 eventos/hora no IAH está associado a um incremento de 9% a 17% no risco de eventos CV. A força dessa associação pode variar conforme subgrupos: o risco é mais pronunciado em homens com menos de 70 anos e em indivíduos com sonolência diurna excessiva. O risco é menor ou não significativo para AOS leve e moderada, sugerindo um efeito dose-resposta, mas com maior impacto nos extremos superiores do IAH.^20^ (ver nota importante 3)

**Figura 1:**
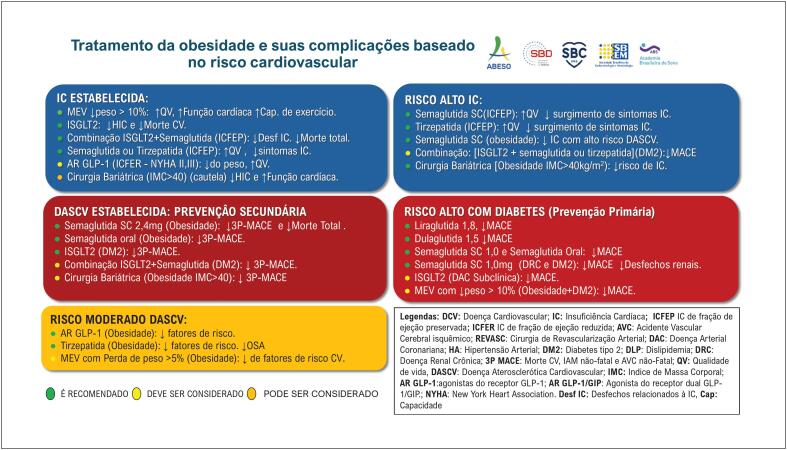
Resumo das intervenções terapêuticas para obesidade e suas complicações em relação às categorias de risco na obesidade.

Conforme apresentado na Figura 1 , o fluxograma fornece um resumo do manejo da obesidade e suas complicações, orientado pela avaliação do risco cardiovascular.

**Table:** 

Nota importante 2: Exames adicionais para IC
Pessoas com pró-peptídeo natriurético tipo B N-terminal (NT-proBNP) e o peptídeo natriurético tipo B (BNP) elevados, ou com sintomas de IC, deverão ser submetidas à investigação diagnóstica complementar. A dosagem de NT-proBNP e o BNP deve ser contextualizada na presença da obesidade, uma vez que níveis mais baixos são observados em comparação a indivíduos sem obesidade.^18^ O limite de NT-proBNP para exclusão de IC (< 125 ng/L) apresenta sensibilidade baixa (67%) em indivíduos com IMC > 35 kg/m^2^. Nestes, um limite de exclusão mais baixo (50 ng/L) mostrou ter maior sensibilidade (93% a 98%). Por outro lado, o limiar de inclusão > 220 ng/mL obteve maior especificidade (82% a 89%).^21^ O uso isolado de NT-proBNP (ou BNP) para orientar o diagnóstico de IC em indivíduos com obesidade deve ser criterioso. Na presença de sintomas e sinais clínicos de IC, mesmo com valores normais, a investigação por métodos diagnósticos complementares é recomendada.^17 , 21^ Em situações como a fibrilação atrial, pode ser necessária a investigação adicional com ecocardiograma, eletrocardiograma ou holter 24h.

**Table:** 

Nota importante 3: Classificação da Apnéia obstrutiva do sono
O Índice de Apneia-Hipopneia (IAH) é a medida mais utilizada para classificar a gravidade da AOS. Ele é calculado somando o número de apneias e hipopneias (reduções no fluxo respiratório) e dividindo pelo número de horas de sono. Normal: IAH menor que 5. Leve: IAH entre 5 e 15. Moderada: IAH entre 15 e 30. Grave: IAH acima de 30.

## PARTE 2 – METAS DE PERDA DE PESO

### 2.1. Meta de Perda de Peso para Redução de Fatores de Risco

**Table:** 

R5.	Grau de recomendação	Nível de evidência
**É RECOMENDADA** a redução sustentada de pelo menos 5% do peso, em indivíduos adultos com sobrepeso ou obesidade e RISCO DASCV MODERADO, para redução de fatores de risco CV, como HAS e dislipidemia, bem como para atrasar ou evitar o surgimento de diabetes tipo 2.	I	A

Sumário de evidências (R5):

- Estudos com modificação de estilo de vida, como o DPP (Diabetes Prevention Program) e o Look AHEAD demonstraram que perdas de peso modestas, de pelo menos 5% do peso corporal, têm impacto sobre redução de fatores de risco cardiometabólicos.^22 , 23^
- O estudo DPP randomizou 3.234 participantes com pré-diabetes ou intolerância à glicose para receberem placebo, metformina (850 mg duas vezes por dia) ou um programa de modificação do estilo de vida, visando perda de peso ≥ 7% e prática de pelo menos 150 minutos de atividade física semanal. A intervenção no estilo de vida reduziu a incidência de diabetes em 58% (IC 95% 48-66%), enquanto a metformina reduziu em 31% (IC 95% 17-43%) em comparação ao placebo após 2,8 anos.^22^
- O estudo Look AHEAD foi um ensaio clínico randomizado (ECR) controlado que avaliou o efeito da intervenção intensiva no estilo de vida ou orientação tradicional e educação em diabetes em 5.145 adultos com sobrepeso ou obesidade (IMC médio = 36 kg/m^2^) e diabetes tipo 2. O desfecho primário foi composto por morte por causas CV, infarto agudo do miocárdio não fatal, acidente vascular cerebral não fatal ou internação hospitalar por angina. O ensaio foi interrompido precocemente por futilidade, após uma mediana de seguimento de 9,6 anos. Embora o desfecho primário não tenha sido atingido, a intervenção intensiva no estilo de vida levou à maior perda de peso (8,6% vs. 0,7% em 1 ano; 6,0% vs. 3,5% no final do estudo) e promoveu maiores reduções na HbA1c e em fatores de risco CV.^23^ A magnitude da perda de peso em 1 ano foi fortemente associada a benefícios na glicemia, pressão arterial, triglicerídeos e colesterol HDL (p < 0,0001), mas não no colesterol LDL (p = 0,79). Em comparação aos participantes com peso estável, os indivíduos que perderam entre 5 a < 10% (7,25 ± 2,1 kg) do peso corporal tiveram maiores probabilidades de atingir redução na HbA1c (OR 3,52 [IC 95% 2,81-4,40]), diminuição de 5 mmHg na pressão arterial diastólica (OR 1,48 [1,20-1,82]), de 5 mmHg na pressão arterial sistólica (OR 1,56 [1,27-1,91]), de 40 mg/dL nos triglicerídeos (2,20 [1,71-2,83]) e aumento de 5 mg/dL no colesterol HDL (OR 1,69 [1,37-2,07]).^24^

### 2.2. Meta de Perda de Peso para Redução de Eventos Cardiovasculares

**Table:** 

R6.	Grau de recomendação	Nível de evidência
**DEVE SER CONSIDERADA** a redução sustentada de pelo menos 10% do peso máximo já atingido na vida, em indivíduos adultos, com sobrepeso ou obesidade com risco DASCV MODERADO/ALTO, para redução de eventos CV.	IIa	B

Sumário de evidências (R6):

- O estudo Da-Qing foi um ECR chinês que avaliou os resultados de 6 anos de intervenção com mudança de estilo de vida em 577 indivíduos com pré-diabetes e sobrepeso (média de IMC = 25,7 kg/m^2^) na incidência de diabetes, eventos CV, complicações microvasculares, morte CV, morte por todas as causas e expectativa de vida. Após 30 anos de seguimento, houve redução nas taxas de eventos CV (HR 0,74, IC 95% [0,59, 0,92]), morte CV (0,67, IC 95% [0,48, 0,94], p = 0,022), morte global (0,74, IC 95% [0,61, 0,89], p = 0,0015) e postergaram em até 4 anos o diagnóstico de diabetes.^25^
- Embora o desfecho primário do estudo LooK-AHEAD não tenha sido atingido, uma avaliação observacional *post-hoc* sugere uma associação entre a magnitude da perda de peso inicial e a redução de eventos CV no longo prazo em pessoas com obesidade e diabetes tipo 2. Ao longo de um seguimento médio de 10,2 anos (IQR 9,5-10,7), o grupo de indivíduos que perdeu pelo menos 10% do peso corporal no primeiro ano do estudo teve um risco 21% menor do desfecho primário (taxa de risco ajustada [HR] 0,79, IC 95% 0,64-0,98; p = 0,034) e um risco 24% menor de desfecho secundário (HR ajustado 0,76, IC 95% 0,63-0,91; p = 0,003) em comparação com indivíduos com peso estável ou ganho de peso. Nas análises que consideraram o grupo controle como grupo de referência, os participantes do grupo de intervenção intensiva no estilo de vida que perderam pelo menos 10% do peso corporal tiveram um risco 20% menor do desfecho primário (HR ajustado 0,80, IC 95% 0,65-0,99; p = 0,039) e 21% menor do desfecho secundário (HR ajustado 0,79, IC 95% 0,66-0,95; p = 0,011).^26 , 27^
- No estudo *Semaglutide Effects on Cardiovascular Outcomes in People With Overweight or Obesity* (SELECT), que demonstrou a superioridade da semaglutida 2,4 mg em relação ao placebo na redução de eventos CV em pessoas com obesidade e DCV estabelecida, a redução de peso associada ao benefício foi de apenas 9%. Análises posteriores sugerem que grande parte dos benefícios sejam independentes da perda de peso, especialmente nas reduções de eventos cardiovasculares maiores (MACE) observadas logo ao início do estudo (ou seja, antes de reduções significativas de peso terem sido atingidas. Entretanto, não se pode descartar que ao menos uma pequena parte desse benefício esteja relacionada à perda de peso.^28^
- As cirurgias bariátricas também demonstraram redução de eventos CV e mortalidade em populações com obesidade. O *Swedish Obese Subjects (SOS) study* , um estudo de coorte prospectivo e não randomizado, avaliou 4.047 indivíduos com obesidade (IMC ≥ 34 kg/m² para homens e ≥ 38 kg/m² para mulheres), dentre os quais 2.010 foram submetidos à cirurgia bariátrica (bandagem gástrica, gastroplastia vertical com anel ou *bypass* gástrico) e 2.037 receberam tratamento convencional para obesidade. O acompanhamento médio foi de 14,7 anos. A perda de peso média observada no grupo cirúrgico após 10 anos foi de 17% (vs. 1% no grupo com terapia convencional). Em relação aos eventos CV combinados (IAM fatal e não fatal, AVC fatal e não fatal, angina de peito e IC), o grupo cirúrgico apresentou uma redução significativa de 33%, além de 53% na morte por DCV (HR 0,47; IC 95% 0,29-0,76; p = 0,002) vs. o grupo de tratamento convencional. Embora não se possa excluir que os benefícios se devam a efeitos próprios da cirurgia, o fato de que mais de 80% dos procedimentos cirúrgicos foram restritivos (gastroplastia vertical em banda e banda gástrica, procedimentos nos quais os efeitos hormonais contribuintes para a perda de peso são menos relevantes), é possível supor que o principal fator associado à redução de MACE tenha sido a perda de peso significativa (> 15%) e sustentada.^29^
- Dessa forma, considerando-se as evidências apresentadas, este painel julgou razoável a recomendação de metas de perda de pelo menos 10% do peso máximo atingido^30^ para pessoas de risco DASCV moderado ou elevado objetivando redução de eventos CV.

### 2.3. Objetivos de Redução Ponderal para Complicações Associadas à Fibrilação Atrial

**Table:** 

R7.	Grau de recomendação	Nível de evidência
**É RECOMENDADA** a redução sustentada de pelo menos 10% do peso em indivíduos com obesidade e Fibrilação Atrial (FA) paroxística ou permanente, para reduzir o risco de complicações relacionadas à FA.	I	B

Sumário de evidências (R7):

- A redução de peso em indivíduos com obesidade tem demonstrado impacto positivo na redução da carga de sintomas e na recorrência da fibrilação atrial (FA).^31^
- Um ECR controlado, unicêntrico, parcialmente cego, realizado na Austrália, com 150 pessoas com obesidade ou sobrepeso e FA assintomática, evidenciou que um programa estruturado de gerenciamento de peso resultou em redução significativa da carga de sintomas, da gravidade e do número de episódios de FA em 15 meses de seguimento. O grupo intervenção perdeu mais peso comparado ao grupo controle (14,3 kg vs. 3,6 kg), e teve maior redução no escore relacionado à severidade dos sintomas, além de maior redução na espessura septal interventricular.^32^
- Outra metanálise revelou que uma perda de pelo menos 10% do peso está associada a uma menor recorrência de FA, redução na carga de FA e melhora na gravidade dos sintomas.^33^ Da mesma forma, perdas ponderais após ablação por cateter reduziram a recorrência de FA após 12 meses de seguimento.^34^
- No estudo SOS, a cirurgia bariátrica reduziu o risco de FA de início recente em comparação ao cuidado usual. A redução de risco foi mais pronunciada nos indivíduos mais jovens e com pressão arterial diastólica elevada.^35^

## Parte 3 – Intervenções para Redução de Eventos e Fatores de Risco

### 3.1. Mudança de Estilo de Vida

**Table:** 

R8.	Grau de recomendação	Nível de evidência
**É RECOMENDADA** a adoção de mudanças de estilo de vida, incluindo dieta saudável e atividade física, para todas as pessoas com sobrepeso ou obesidade, independentemente do risco CV, com o objetivo de reduzir o peso, melhorar a saúde, a qualidade de vida e prevenir HAS, diabetes tipo 2, DLP, DASCV e IC.	I	A

Sumário de evidências (R8):

- A mudança de estilo de vida (MEV) no indivíduo com sobrepeso ou obesidade deve incluir um programa alimentar com distribuição adequada de macronutrientes em conjunto com a prática de exercício físico aeróbico e resistido.^36^
- A abordagem da MEV deverá ser multiprofissional, com equipe composta por nutricionista, educador físico e psicólogo, e conduzida em encontros individuais ou em grupo. A incorporação da MEV não deverá postergar o início da terapia antiobesidade, quando esta estiver indicada.^37^
- O aconselhamento nutricional deverá focar na redução do tamanho das porções, no aumento da ingestão de frutas, verduras e hortaliças, e na redução de consumo de álcool e alimentos ultraprocessados. Além disso, deve visar um déficit energético inicial de 500-750 kcal/dia, que precisará ser ajustado para o peso corporal e atividades individuais.^37^
- Ensaios clínicos randomizados (ECRs) que avaliaram medicamentos com efeito moderado na perda de peso, como sibutramina,^38^ liraglutida^39^ e a combinação bupropiona + naltrexona,^40^ demonstraram que a combinação com MEV apresentou melhores resultados em relação à redução de peso corporal e de fatores de risco cardiometabólicos.
- Com a disponibilidade de fármacos antiobesidade de maior potência como a semaglutida^41^ e a tirzepatida,^42^ déficits calóricos mais expressivos podem ser alcançados. Neste contexto, o consumo variado de macronutrientes, especialmente proteínas, deverá ser monitorado para evitar a sarcopenia e as deficiências nutricionais, e permitir o estabelecimento de um hábito alimentar saudável e sustentável.^43^
- Da mesma forma, este objetivo será alcançado com padrões alimentares ricos em alimentos *in natura* e minimamente processados, como aqueles descritos nas dietas Mediterrânea e DASH ( *Dietary Approach to Stop Hipertension)* , incluem cereais e grãos integrais, frutas e verduras, carnes brancas e magras e fontes de proteínas vegetais, como leguminosas e oleaginosas.^44 , 45^
- Tais padrões alimentares demonstraram bons resultados em relação à melhora de risco cardiometabólico^45^ e podem ser utilizados como referência, mas adaptações às preferências alimentares, aspectos socioeconômicos e culturais brasileiros devem ser realizadas, para melhor adesão.^46^
- O consumo de alimentos ultraprocessados, ricos em gorduras saturadas e açúcares refinados deve ser limitado pela associação com piora da composição corporal e aumento de mortalidade global e CV.^47^

### 3.2. Manejo da Farmacoterapia para Redução de Eventos e Fatores de Risco

#### 3.2.1. Obesidade e Risco de DCVA Moderado ou Alto

**Table:** 

R9.	Grau de recomendação	Nível de evidência
**É RECOMENDADO** o tratamento com um agonista do receptor GLP-1 (AR GLP-1) ou coagonista dos receptores GLP-1/GIP, para indivíduos adultos com sobrepeso ou obesidade e risco DASCV MODERADO ou ALTO, para redução de peso e fatores de risco CV.	I	A

Sumário de evidências (R9):

- A liraglutida, agonista do receptor peptídeo-1 semelhante ao glucagon (AR GLP-1) com eficácia na perda de peso na dose de 3,0 mg/dia, teve seus efeitos no tratamento da obesidade e suas complicações avaliados no programa SCALE ( *Satiety and Clinical Adiposity – Liraglutide Evidence* ). O estudo SCALE Obesidade e PreDiabetes randomizou 2.254 pacientes para receber liraglutida 3,0 mg ou placebo. Após 56 semanas, 63,2% e 33,1% dos pacientes perderam, respectivamente, mais de 5% e 10% do peso inicial. Após 3 anos, nos pacientes com pré-diabetes, o risco de desenvolver diabetes foi reduzido em 79%; os pacientes em uso de liraglutida levaram 2,7 vezes mais tempo para desenvolver diabetes vs. placebo.^48^
- Uma análise *post hoc* utilizando dados agrupados de 5.908 participantes dos 5 ERC do programa SCALE (liraglutida vs. placebo ou orlistat) demonstrou a segurança CV da liraglutida 3,0 mg em pessoas com obesidade.^49^
- O STEP 1 ( *Semaglutide Treatment Effect in People with obesity* ) incluiu 1.961 pacientes com sobrepeso e/ou obesidade sem diabetes tipo 2, seguidos por 68 semanas. Todos os participantes receberam dieta hipocalórica, com déficit de 500 Kcal por dia, e orientação para praticarem 150 minutos de atividade física por semana. Ao final do estudo, os participantes do grupo semaglutida 2,4 mg perderam 16,9% do peso, com nadir ao redor da semana 60, enquanto os do grupo placebo perderam 2,4%.^50^
- A tirzepatida também se mostrou eficaz na redução do risco de progressão para diabetes em pacientes com obesidade e pré-diabetes. Em uma análise do estudo SURMOUNT-1, com 1.032 participantes com obesidade e pré-diabetes tratados com tirzepatida por aproximadamente 3 anos (176 semanas), houve menor incidência de diabetes tipo 2 vs. o grupo placebo (1,3% vs. 13,3%; razão de risco 0,07 [IC 95% 0,0-0,1; p < 0,001). Adicionalmente, após 17 semanas de suspensão da tirzepatida, 2,4% do grupo randomizado para tirzepatida vs. 13,7% do grupo placebo desenvolveram diabetes tipo 2 (razão de risco 0,12, IC 95% 0,1-0,2; p < 0,001). Em termos absolutos, quase 99% das pessoas com pré-diabetes que receberam tirzepatida permaneceram sem diabetes. Os percentuais médios de perda de peso dos grupos tirzepatida 5 mg, 10 mg e 15 mg foram de −12,3%, −18,7% e −19,7%, respectivamente, vs. −1,3% no grupo placebo após 3 anos (p < 0,001 vs. placebo para todas as comparações). Ademais, uma perda de peso superior a 20% se associou a uma razão de risco de progressão para diabetes tipo 2 de 0,07, com um NNT de 9 para prevenir um caso de diabetes, e com 92% dos pacientes apresentando reversão para normoglicemia, reforçando a importância da perda de peso na prevenção de diabetes.^51^
- Em relação aos indivíduos com obesidade e diabetes, uma revisão sistemática avaliou o efeito das medicações antidiabéticas não-insulínicas na perda de peso de pacientes com diabetes tipo 2 em diversos ECRs. Liraglutida, semaglutida e tirzepatida resultaram em maior perda de peso em comparação às outras classes terapêuticas (perdas acima de 5%).^52^

**Table:** 

R10.	Grau de recomendação	Nível de evidência
**PODE SER CONSIDERADO** o uso de outras medicações antiobesidade com comprovada eficácia e segurança CV, em indivíduos adultos com sobrepeso ou obesidade e risco DASCV MODERADO ou ALTO, na impossibilidade de iniciar ou manter o tratamento com um AR GLP-1 ou coagonista dos receptores GLP-1/GIP, para redução de fatores de risco CV (ver Tabela 4 ).	IIb	B

Sumário de evidências (R10):

- O orlistate é um inibidor da lipase gástrica e pancreática, que promove redução de peso através da redução de absorção em 30% da gordura ingerida. O estudo XENical in the prevention of diabetes in obese subjects (XENDOS) randomizou 3.305 indivíduos com obesidade (IMC ≥ 30 kg/m²) e glicemia normal (79%) ou tolerância à glicose diminuída (21%) para orlistate (120 mg 3x/dia) ou placebo, ambos associados a MEV. Após 4 anos de tratamento, a incidência cumulativa de diabetes foi de 9% no grupo placebo vs. 6,2% no grupo orlistate, o que corresponde a uma redução de risco de 37,3% (p = 0,0032). Uma análise exploratória demonstrou que a maior perda de peso foi o principal fator determinante para a prevenção do diabetes. Ao longo dos 4 anos, os indivíduos que usaram orlistate perderam mais peso em relação ao placebo (5,8 versus 3,0 kg, respectivamente; p < 0,001).^53^
- Na metanálise que incluiu quatro ECRs avaliando a eficácia da combinação de naltrexona/bupropiona vs. placebo em um ano, a diferença de perda de peso foi de 5,0 kg (5.94-3.96). Em comparação com o placebo, 55% (48-61%) dos pacientes em uso da medicação atingiram pelo menos 5% de perda de peso, e 30% (24-37%) atingiram pelo menos 10% de perda de peso.^54^ No estudo COR-Diabetes ( *Contrave Obesity Research-Diabetes* ), que avaliou pacientes com diabetes tipo 2, os indivíduos tratados tiveram uma redução média de −11,2% nos triglicérides (vs. −0,8% no grupo placebo) e um aumento de 3,0 ± 0,5 mg/dL no colesterol HDL (vs. −0,3 ± 0,6 mg/dL no grupo placebo), sem efeito significativo no colesterol LDL.^55^
- A segurança CV da naltrexona/bupropiona foi testada no estudo LIGHT (n = 4.454), que foi interrompido prematuramente após a liberação ao público de dados interinos confidenciais. Porém, atingiu-se 50% do número de eventos pré-planejados, e MACE ocorreram em 102 pacientes (2,3%) no grupo placebo e 90 pacientes (2,0%) do grupo naltrexona/bupropiona (HR, 0,88; HR ajustado para o IC 99,7%, 0,57-1,34). Assim, houve demonstração de segurança num período médio de dois anos, com manutenção da perda de peso atingida.^56^ Uma revisão sistemática e metanálise publicada posteriormente também sinalizou a segurança CV da medicação.^57^
- Os medicamentos aprovados no Brasil para o tratamento da obesidade que apresentaram perda de peso e segurança CV superiores quando comparados ao placebo estão listados na Tabela 4 . Os eventos adversos e contraindicações mais comumente observados estão listados na Tabela 5 .^58^

**Tabela 4:** Resumo dos principais efeitos dos medicamentos antiobesidade aprovados no Brasil

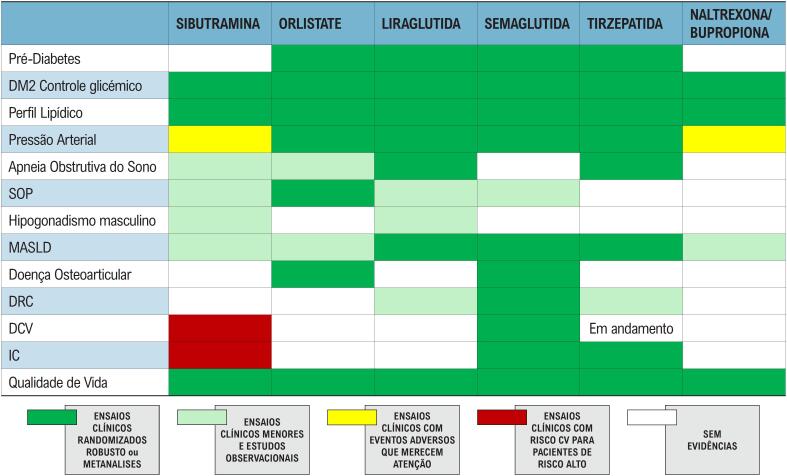

* Excluídos estudos destinados primariamente ao tratamento do diabetes. Adaptado de Moreira et al.^58^

Adaptado de Moreira et al.^58^

**Tabela 5:** Principais efeitos colaterais das medicações aprovadas para o tratamento da obesidade

Medicação e dose	Mais de 10% dos pacientes	Efeitos específicos que merecem atenção
Sibutramina (10–15 mg)	Constipação, xerostomia, insônia	Taquicardia/aumento da frequência cardíaca, aumento da pressão arterial, cefaleia, ansiedade
Orlistate (120 mg, 3x/dia)	Diarreia/esteatorreia/urgência, flatulência, infecções do trato respiratório superior/gripe, cefaleia, hipoglicemia	Reações de hipersensibilidade, deficiência de vitaminas lipossolúveis no longo prazo
Liraglutida (3,0 mg/dia)	Náusea e vômito, diarreia, constipação	Reações no local da injeção, aumento da frequência cardíaca, insônia, colelitíase, astenia e fadiga, hipoglicemia
Semaglutida (2,4 mg/semana)	Náusea e vômito, diarreia, constipação, dor abdominal, cefaleia, fadiga	Reações no local da injeção, aumento da frequência cardíaca, colelitíase, hipoglicemia
Tirzepatida (10 e 15 mg/semana)	Hipoglicemia (quando utilizada com sulfonilureias ou insulina), náuseas, diarreia	Reações de hipersensibilidade, aumento da frequência cardíaca, reações no local da injeção
Bupropiona + Naltrexona (360/32 mg/dia)	Náusea, constipação, cefaleia, vômito	Pensamentos ou ações suicidas, convulsões, risco de dose excessiva com opioides, retirada repentina de opioides, reações alérgicas severas, aumento da pressão arterial ou da frequência cardíaca, hepatite, episódios de mania, glaucoma de ângulo fechado, hipoglicemia (quando utilizada com sulfonilureias ou insulina), síndrome serotoninérgica

Adaptado de Moreira et al.^58^

#### 3.2.2. Obesidade e Risco de DCVA Estabelecido

**Table:** 

R11.	Grau de recomendação	Nível de evidência
**É RECOMENDADO** o tratamento com semaglutida SC 2,4mg em indivíduos com IMC ≥27Kg/m², sem diabetes, e com doença CV estabelecida (PREVENÇÃO SECUNDÁRIA) para redução de morte CV, IAM e AVC ( Figura 1 )	I	B

Sumário de evidências (R11):

- O estudo SELECT foi um ECR destinado a avaliar a prevenção secundária de eventos CV que incluiu 17.604 adultos com sobrepeso ou obesidade, com idade média de 61,6 anos e IMC de 33,34 kg/m^2^. A população estudada não apresentava diagnóstico prévio de diabetes, e tinha como critério de inclusão a presença de doença CV estabelecida. Uma perda de peso de 9% do peso original foi obtida, em média. Os resultados mostraram uma redução de 20% em MACE (morte CV, IAM não fatal e AVC não fatal) (6,5% versus 8,0% com placebo, HR 0,80, IC 95% 0,72-0,90, p < 0,001).^59^

**Table:** 

R12.	Grau de recomendação	Nível de evidência
**NÃO É RECOMENDADO** o uso de sibutramina em pacientes com obesidade e RISCO DASCV ALTO.	III	B

Sumário de evidências (R12):

- O estudo SCOUT avaliou o uso de sibutramina versus placebo em pessoas com sobrepeso/obesidade, com DCV prévia e/ou diabetes tipo 2 mais um fator de risco CV. O risco de um evento de desfecho primário (infarto do miocárdio não fatal, acidente vascular cerebral não fatal, ressuscitação após parada cardíaca ou morte CV) foi de 11,4% no grupo da sibutramina, em comparação com 10,0% no grupo do placebo (razão de risco, 1,16; IC 95%, 1,03 a 1,31; p = 0,02). Dessa forma, este painel considera que a utilização de sibutramina em pessoas com obesidade e risco DASCV alto ou DAC crônica não é recomendado.^60^

#### 3.2.3. Obesidade com Diabetes Tipo 2

**Table:** 

R13.	Grau de recomendação	Nível de evidência
**É RECOMENDADO** o tratamento farmacológico com um AR GLP-1 (Liraglutida, Dulaglutida ou Semaglutida SC ou Oral) em pessoas com Diabetes tipo 2, obesidade ou sobrepeso e RISCO DASCV ALTO, para redução de EVENTOS CV.	I	A

Sumário de evidências (R13):

- Cinco ECRs de desfechos CV – LEADER, SUSTAIN-6, REWIND, HARMONY, AMPLITUDE-O e SOUL demonstraram, de forma consistente, a eficácia e segurança dos AR GLP-1 em pacientes com diabetes tipo 2, além de evidenciarem um efeito preventivo secundário desses fármacos em indivíduos com diabetes tipo 2 e DCV.^61^
- Uma revisão sistemática com metanálise de ECRs em pacientes com diabetes tipo 2 demonstrou que os AR GLP-1 promovem uma redução significativa de 14% no desfecho composto MACE (morte CV, IAM não-fatal e AVC não-fatal), além de diminuir a mortalidade por todas as causas em 12%. Os AR GLP-1 reduziram igualmente as hospitalizações por IC em 11% e os desfechos renais compostos em 21%. Notavelmente, esses benefícios clínicos ocorrem sem aumento do risco de hipoglicemia grave, retinopatia ou eventos adversos pancreáticos, reforçando o perfil de segurança desses fármacos no manejo da obesidade e do diabetes tipo 2.^62^
- Uma revisão sistemática com metanálise em rede demonstrou que os AR GLP-1 promovem redução significativa da mortalidade por todas as causas e de mortalidade CV, além de diminuir a incidência de IAM, AVC não-fatais, insuficiência renal e hospitalizações por IC em indivíduos com diabetes tipo 2.^63^
- O estudo SOUL, ECR duplo-cego e controlado por placebo, avaliou a eficácia CV do semaglutida oral em 9.650 pacientes com diabetes tipo 2 e DASCV, doença renal crônica (DRC) ou ambas. Após um acompanhamento médio de 47,5 meses, o uso da semaglutida oral reduziu significativamente o risco de MACE, incluindo morte por causas CV, IAM não-fatal e AVC não-fatal. A incidência desses eventos foi de 3,1 por 100 pessoas-ano no grupo semaglutida versus 3,7 por 100 pessoas-ano no grupo placebo, resultando em uma redução do risco relativo de 14% (HR 0,86; IC 95%: 0,77-0,96; p = 0,006).^64^

#### 3.2.4. Obesidade com Diabetes Tipo 2 e Doença Renal Crônica

**Table:** 

R14.	Grau de recomendação	Nível de evidência
**DEVE SER CONSIDERADO** o uso de semaglutida 1,0 mg em pacientes com obesidade ou sobrepeso, diabetes tipo 2 e DRC com taxa de filtração glomerular ≥ 25ml/min/1,73m^2^, para redução de eventos cardiorrenais.	IIa	B

Sumário de evidências (R14):

- O estudo FLOW ( *Effect of semaglutide versus placebo on the progression of renal impairment in people with type 2 diabetes and chronic kidney disease* ) é um estudo multicêntrico que incluiu 3.534 participantes com diabetes tipo 2, DRC e sobrepeso/obesidade para investigar o efeito do tratamento com semaglutida na dose de 1,0 mg via subcutânea semanal na progressão da doença renal. O desfecho primário composto foi o declínio persistente da TFGe ≥ 50% desde o início do estudo, doença renal terminal, morte por doença renal ou morte por DCV. O estudo foi interrompido precocemente por eficácia. O estudo alcançou redução significativa da progressão da doença renal, bem como da mortalidade CV e renal de 24% para as pessoas tratadas com semaglutida 1,0 mg. Além disso, a semaglutida 1,0 mg apresentou impacto positivo em outros desfechos clínicos: 21% de redução no risco de morte por causas CV (HR 0,71; IC 95%: 0,56-0,89), 21% de redução nos desfechos renais compostos (HR 0,79; IC 95%: 0,66-0,94), 18% de redução no risco de eventos CV graves (HR 0,82; IC 95%: 0,68-0,98; p = 0,029) e 20% de redução na mortalidade por todas as causas (HR 0,80; IC 95%: 0,67-0,95; p = 0,01).^65^

**Table:** 

NOTA IMPORTANTE 4 Uso da semaglutida na DRC
O uso de semaglutida pode ser considerado quando: TFG ≥ 50-75ml/min/1,73m^2^ e RAC > 300 a < 5000mg/g. TFG 25-50 e RAC >100 e < 5000mg/g. TFG entre 15 e 25ml/min/1,73m^2^. Usar com cautela, pois não há evidências. Deve ser evitado quando TFG < 15ml/min/1,73m^2^.

#### 3.2.5. Obesidade com Apneia Obstrutiva do Sono (AOS)

**Table:** 

R15.	Grau de recomendação	Nível de evidência
**É RECOMENDADA** a perda de peso para indivíduos com obesidade e apneia obstrutiva do sono moderada a grave, para melhora ou remissão da apneia.	I	C

Sumário de evidências (R15):

- As evidências sobre o impacto do tratamento da obesidade sobre a gravidade e remissão da apneia obstrutiva do sono tem ganhado crescente atenção, mas ainda apresentam limitações significativas.^66^
- Em termos de medidas não medicamentosas, estudos randomizados pequenos e com seguimento curto sugerem que estratégias interdisciplinares visando a redução de peso podem reduzir a gravidade da apneia obstrutiva do sono, especialmente nos casos mais leves.^67 , 68^
- A remissão (normalização do índice de apneia sem a necessidade de tratamentos específicos tais como o CPAP( *Continuous Positive Airway Pressure* ) pode ocorrer em alguns casos. Importante notar que, para alcançar o resultado, alguns dos estudos adotaram medidas muito restritivas de intervenção, difíceis de serem amplamente implementadas a longo prazo.^69^

**Table:** 

R16.	Grau de recomendação	Nível de evidência
**PODE SER CONSIDERADO** o uso de liraglutida em combinação com mudança de estilo de vida em indivíduos com obesidade e apneia obstrutiva do sono moderada a grave, para redução da gravidade da AOS.	IIb	B

Sumário de evidências (R16):

- Em relação ao tratamento farmacológico da obesidade, as evidências derivadas de estudos randomizados são limitadas a dois estudos até o momento com duas medicações testadas (liraglutida e tirzepatida).^70 , 71^
- O estudo SCALE testou a liraglutida na dose de 3,0mg por dia por 32 semanas em pacientes com obesidade sem DM, que tinham AOS moderada ou grave e não usavam, ou não se adaptaram ao uso de CPAP adjuvante à dieta e exercícios. Após 32 semanas, a redução média do IAH foi maior com liraglutida do que com placebo (-12,2 vs. −6,1 eventos/ h) em paralelo à maior porcentagem média de perda de peso da liraglutida em comparação com o placebo (-5,7% vs. −1,6%). Este estudo mostrou que a IAH residual foi relevante, sugerindo que os pacientes em geral não tiveram remissão da apneia obstrutiva do sono.^70^

**Table:** 

R17.	Grau de recomendação	Nível de evidência
**DEVE SER CONSIDERADO** o uso de **tirzepatida** em combinação com mudança de estilo de vida em indivíduos com obesidade e apneia obstrutiva do sono moderada a grave, para redução da gravidade ou remissão da AOS.	IIa	B

Sumário de evidências (R17):

- O estudo SURMOUNT-OSA foi um ECR multicêntrico, com 469 pacientes com obesidade e apneia moderada a grave, com ou sem uso prévio de CPAP, os quais foram randomizados para tratamento com tirzepatida ou placebo. Em comparação ao placebo, o tratamento com tirzepatida nas doses de 10 a 15 mg por semana, durante 52 semanas, resultou em uma redução percentual de peso de 16 a 17% em ambos os subestudos (com ou sem uso prévio de CPAP). A perda de peso foi acompanhada por uma redução no IAH de 20 e 24 eventos por hora em comparação ao placebo, e uma redução relativa de eventos, respectivamente de 48 e 56%, em pacientes com e sem CPAP. Uma proporção significativa atingiu remissão da apneia obstrutiva do sono ou uma apneia dita "não relevante" (leve ou sem sintomas).^71^

**Table:** 

NOTA IMPORTANTE 5: Tirzepatida na AOS
O ensaio clínico SURMOUNT-OSA, levou a *Food and Drug Administration* (FDA) a aprovar a tirzepatida como o primeiro medicamento para o tratamento da apneia obstrutiva do sono moderada a grave em adultos com obesidade, para ser usada em combinação com uma dieta hipocalórica e aumento da atividade física.^72^ O benefício da tirzepatida na redução da gravidade da Apneia Obstrutiva do Sono, na redução do peso e de marcadores cardiometabólicos ocorreu em pacientes usando ou não a pressão positiva contínua de vias aéreas superiores (CPAP). Estudos randomizados e metanálises mostram que o tratamento da AOS *per se* com CPAP não promove redução do peso. Desta forma, medidas adicionais para redução do peso precisam ser implementadas.^73^

#### 3.2.6. Obesidade com Insuficiência Cardíaca

**Table:** 

R18.	Grau de recomendação	Nível de evidência
**É RECOMENDADA** a redução de peso em pessoas com obesidade e IC estabelecida para melhorar a qualidade de vida, a função cardíaca e a capacidade para o exercício.	I	A

Sumário de evidências (R18):

- Uma metanálise de 19 ECRs e estudos observacionais, envolvendo 449.882 pacientes com obesidade, mostrou que a perda de peso, apesar de não reduzir mortalidade, melhora a qualidade de vida, a função ventricular e a capacidade para o exercício.^74^
- Uma metanálise de 29 estudos mostrou que a perda de peso intencional através de intervenções como a cirurgia bariátrica, pode levar à melhora na função cardíaca e na qualidade de vida em pacientes com IC e obesidade. A cirurgia bariátrica foi associada a uma redução no risco de desenvolver IC e melhora na função diastólica e na massa ventricular esquerda. Uma curva em J foi observada entre índice de massa corporal (IMC) e risco de IC com o risco máximo na obesidade grave (IMC > 40 kg/m^2^) de 1,73 (IC 95% 1,30- 2,31), p < 0,001). Embora o paradoxo da obesidade tenha sido observado para mortalidade por todas as causas, o grupo com sobrepeso foi associado à menor mortalidade CV (OR: 0,86 (IC 95% 0,79 a 0,94), sem diferença significativa entre outras categorias de IMC. A perda de peso induzida pela cirurgia bariátrica em pessoas com obesidade sem IC estabelecida, fibrilação atrial ou DAC conhecida, foi associada com redução da massa do ventrículo esquerdo (p < 0,0001), melhora na função diastólica do ventrículo esquerdo (p ≤ 0,0001) e redução do tamanho do átrio esquerdo (p = 0,02).^75^

**Table:** 

R19.	Grau de recomendação	Nível de evidência
**É** **RECOMENDADO** o uso da semaglutida SC (2,4 mg/semana) ou da tirzepatida (5-15mg/semana) em pacientes com obesidade (IMC ≥ 30kg/m²) e IC estabelecida de fração de ejeção preservada (ICFEp), para redução de peso, melhora de qualidade de vida e de sintomas relacionados à IC.	I	A

Sumário de evidências (R19):

- Em pacientes com ICFEp estabelecida, 3 ensaios clínicos mostraram eficácia na resolução de desfechos relacionados à IC.
- Dois ECRs avaliaram o uso de semaglutida 2,4 mg uma vez por semana em indivíduos com IC (ICFEp) e obesidade, demonstrando que o uso do agonista de GLP1 melhorou os sintomas relacionados à IC, capacidade funcional e o peso corporal.^76 , 77^
- O estudo STEP-HFpEF foi um ECR para comparação da semaglutida SC 2,4 mg vs. placebo em 529 indivíduos com obesidade, IC classe funcional II a IV da classificação da *New York Heart Association* (NYHA), níveis elevados de peptídeo natriurético (com limiares estratificados de acordo com o IMC no início do estudo), fração de ejeção do ventrículo esquerdo > 45% e evidência de anormalidades ecocardiográficas. A maioria dos 529 participantes (84%) tinha fração de ejeção do ventrículo esquerdo ≥ 50%. O tratamento com semaglutida 2,4 mg semanal durante 1 ano diminuiu significativamente o peso corporal (perda de 13,3% vs. 2,6% no grupo placebo) e melhorou o escore do resumo clínico do Kansas City Cardiomyopathy Questionnaire Clinical Summary Score (KCCQ) e a distância percorrida em 6 minutos. A diminuição nos níveis de NT-proBNP encontrada foi aproximadamente 15% maior com semaglutida do que no grupo tratado com placebo.^76^
- O STEP-HFpEF DM comparou a semaglutida 2,4 mg SC contra placebo em pessoas com obesidade e diabetes tipo 2. Os resultados seguiram a mesma direção do STEP-HFpEF: a semaglutida levou a maiores reduções nos sintomas relacionados à IC, de limitações físicas, e à maior perda de peso, após 1 ano tratamento.^77^
- O SUMMIT foi um ECR com duração de 104 semanas para avaliação da tirzepatida (titulada até 15 mg SC semanalmente; n = 364) vs. placebo (n = 367) em pacientes com IC classe II a IV, fração de ejeção ≥ 50% e IMC ≥ 30 kg/m². O agravamento dos eventos relacionados à IC ocorreu em 29 pacientes no grupo tirzepatida (8,0%) e em 52 pacientes no grupo placebo (14,2%) (HR, 0,54; IC 95% 0,34-0,85). A morte por causas CV ocorreu em 8 pacientes (2,2%) e 5 pacientes (1,4%), respectivamente (HR, 1,58, IC 95% 0,52-4,83). O tratamento com tirzepatida reduziu o desfecho de morte CV ou agravamento da IC em relação ao placebo e melhorou as condições de saúde neste subgrupo de pacientes (escore clínico KCCQ, distância percorrida em 6 minutos de caminhada, índice de estado de saúde EQ-5D-5L e escore de Impressão Global de Gravidade do Paciente em Saúde Geral)^78^

**Table:** 

R20.	Grau de recomendação	Nível de evidência
**É RECOMENDADO** o uso de inibidores do **SGLT2** (iSGLT2) em pacientes com sobrepeso/obesidade e IC estabelecida (independentemente da fração de ejeção do VE), para redução de hospitalização e morte CV.	I	A

Sumário de evidências (R20):

- Uma metanálise pré-especificada dos estudos DELIVER e EMPEROR-Preserved (n = 12.251) demonstrou que os inibidores do SGLT2 (iSGLT2) reduzem significativamente o risco de morte CV ou hospitalização por IC em pacientes com fração de ejeção preservada ou levemente reduzida (HR 0,80; IC 95% 0,73-0,87).^79^ Ao incluir os estudos DAPA-HF, EMPEROR-Reduced (fração reduzida) e SOLOIST-WHF (fração variada), a análise com 21.947 pacientes confirmou reduções em morte CV ou hospitalização por IC: HR 0,77 (IC 95% 0,72-0,82); morte CV: HR 0,87 (IC 95% 0,79-0,95); primeira hospitalização por IC: HR 0,72 (IC 95% 0,67–0,78) e morte por todas as causas: HR 0,92 (IC 95% 0,86-0,99). Os benefícios foram consistentes em todos os subgrupos, incluindo diferentes faixas de fração de ejeção.

**Table:** 

R21.	Grau de recomendação	Nível de evidência
**PODE SER CONSIDERADO** o uso de AR GLP-1 em pacientes com obesidade e IC com fração de ejeção reduzida (ICFEr), para redução do peso, com exceção da IC em classe IV (NYHA).	IIb	B

Sumário de evidências (R21):

- Em pacientes com IC com fração de ejeção reduzida (ICFEr), as evidências para o tratamento da obesidade com AR GLP-1 são insuficientes e sua segurança ainda é discutida.
- No estudo FIGHT, pacientes com hospitalização recente por ICFEr (FE média de 27%) randomizados para liraglutida apresentaram um aumento numérico, mas não significativo, nas hospitalizações por IC.^80^
- Uma análise *post-hoc* do mesmo estudo entre os pacientes utilizando liraglutida em pelo menos uma visita de acompanhamento e com desfecho em termos de redução de peso, evidenciou uma redução significativa e segura de peso nesta população (-4,1 libras, equivalente a aproximadamente −1,96 kg). A população tinha uma idade mediana de 61 anos, 21% eram do sexo feminino e 69% apresentavam classe funcional NYHA III ou IV; fração de ejeção mediana de 25% (percentil 25-75 19-32%).^81^
- No estudo LIVE, que incluiu pacientes com ICFEr crônica alocados para liraglutida, também houve risco aumentado de eventos cardíacos adversos, embora tenha havido apenas uma morte e uma hospitalização por IC. Cabe ressaltar que o número total de eventos cardíacos adversos foi baixo (12 [10%] com liraglutida vs. 3 [3%] com placebo, p = 0,04).^82^
- Importante destacar que nenhum dos estudos (FIGHT ou LIVE) teve por objetivo o tratamento da ICFEr em pessoas com obesidade, e que a liraglutida não foi utilizada em doses para obesidade.
- Em uma análise *post-hoc* do estudo SELECT, incluindo 1.347 pacientes com ICFEr (IMC médio de 33,4 kg/m²), a semaglutida reduziu o risco de MACE em 35% e do composto de morte CV ou hospitalização ou visita hospitalar de urgência por IC em 21%, embora o efeito apenas nas hospitalizações por IC não tenha sido significativo (HR 1,08; p = 0,11). Deve ser destacado que aproximadamente 60% dos pacientes incluídos apresentavam classe funcional NYHA II e pacientes com IC classe funcional NYHA IV foram excluídos. Além disso, a taxa de eventos adversos durante o acompanhamento foi baixa.^83^
- Em uma análise pré-especificada dos estudos STEP-HFpEF e STEP-HFpEFDM, os efeitos da semaglutida nos desfechos primários e no peso corporal foram semelhantes entre os 3 grupos avaliados com base nos diferentes graus de fração de ejeção inicial (45-49%, 50-59% e ≥ 60%). Da mesma forma, a fração de ejeção de VE não influenciou o resultado obtido com a semaglutida nos seguintes desfechos secundários confirmatórios: distância percorrida nos 6 minutos (p de interação = 0,19), desfecho composto hierárquico (p de interação = 0,43) e proteína C-reativa ultrassensível.^84^ Apesar desses resultados, deve-se observar que todos os AR GLP-1, incluindo a semaglutida, estão associados a um discreto aumento da frequência cardíaca (3-5 bpm).^85^

**Table:** 

NOTA IMPORTANTE 6: Perda de peso em pacientes com ICFEr
Não há ECRs avaliando a perda de peso em pacientes com obesidade e ICFEr nas classes funcionais III-IV, em termos de melhora de sobrevida. Nesses pacientes, a recomendação de mudança de estilo de vida que inclua restrição calórica deve ser realizada com monitorização clínica cuidadosa, pelo risco potencial de piora do estado catabólico, habitualmente observado nos estágios mais avançados da ICFEr, bem como o desenvolvimento de caquexia e consequente aumento da mortalidade. Mais estudos são necessários para avaliar a segurança do uso de medicações antiobesidade em indivíduos com ICFEr classe funcional NYHA III ou IV, em especial as de alta potência (semaglutida 2,4 mg e tirzepatida 15 mg).

#### 3.2.7. Obesidade em Indivíduos com Alto Risco de Insuficiência Cardíaca

**Table:** 

R22.	Grau de recomendação	Nível de evidência
**É RECOMENDADO** o uso da semaglutida SC (2,4 mg/semana) ou da tirzepatida (10-15 mg/semana) em indivíduos com obesidade grau 3 (IMC ≥ 40kg/m²) com risco IC alto, para melhora de qualidade de vida e prevenção do surgimento de sintomas relacionados à IC.	I	C

Sumário de evidências (R22):

- Com base em opinião de especialistas, este painel recomenda o uso semanal tanto da semaglutida 2,4 mg como da tirzepatida 10-15 mg para potencial prevenção de desfechos relacionados à IC em pacientes com obesidade grau 3 ou em risco IC alto. O painel baseou-se nos estudos em IC estabelecida^76 , 78^ e na plausibilidade de haver benefícios nessa população, uma vez que a IC é um processo fisiopatológico contínuo que evolui por fases interligadas mediadas por fatores de risco, dentre os quais a obesidade exerce papel essencial.^17^

**Table:** 

R23.	Grau de recomendação	Nível de evidência
**DEVE SER CONSIDERADA** a combinação de iSGLT2 com AR GLP-1 em pacientes com obesidade, diabetes tipo 2 e risco alto de IC, para redução adicional de desfechos relacionados à ICFEp, por estar associada a maiores reduções, comparativamente à monoterapia com qualquer um dos agentes.	IIa	B

Sumário de evidências (R23):

- Estudos de vida real, metanálises de ensaios clínicos randomizados e estudos observacionais e estudos retrospectivos apontam para um potencial efeito aditivo da combinação iSGLT2/AR GLP-1 sobre a monoterapia com cada um dos agentes. Este efeito, entretanto, ainda precisa ser confirmado em ensaios clínicos randomizados. Este painel avaliou, por opinião de especialistas, que há plausibilidade no efeito aditivo desta associação, uma vez que atuam por mecanismos diferenciados e com potencial para serem aditivos. Como os iSGLT2 são considerados pilares de tratamento da IC e a perda de peso é necessária nos pacientes com obesidade e IC, este painel sugere considerar o uso associado para pacientes com IC ou em risco de IC.
- Uma revisão sistemática com metanálise sugere que os benefícios cardiorrenais podem ser ampliados com a terapia combinada em comparação com a monoterapia. O estudo avaliou o efeito cardiorrenal de combinar o iSGLT2 com o AR GLP-1, comparando com monoterapia com ambas as classes de agentes em pacientes com diabetes tipo 2. Estudos elegíveis foram ECRs e observacionais que compararam iSGLT2 ou AR GLP-1 combinados ou em monoterapia. Foram encontrados 5 ECRs e 10 análises *post hoc* de estudos observacionais. Comparado com a monoterapia com AR GLP-1, a terapia combinada com iSGLT2 e AR GLP-1 foi associada a menor risco de desfechos relacionados à IC (RR 0,63, IC 95% 0,51-0,77, p < 0,001) e mortalidade por todas as causas (RR 0,66, IC 95% 0,50-0,88, p = 0,004) em pacientes com diabetes tipo 2.^86^
- O estudo de vida-real retrospectivo realizado a partir de um banco de dados espanhol, incluiu informações de 15.549 pacientes com diabetes tipo 2, de 2018 a 2022, sendo 46% com obesidade, 71% com hipertensão arterial, 15% com DAC e 10% com IC estabelecida. Três grupos foram estabelecidos de acordo com a terapia em uso: 1) iSGLT2 em monoterapia (n = 12.029; média de uso: 14 meses), 2) AR GLP-1 em monoterapia (n = 1.071; média de uso: 17 meses) ou 3) AR GLP-1 + iSGLT2 (n = 2.449) (média de uso: 14 meses). Os dados foram submetidos a análise por escore de propensão 1:1. A mediana de seguimento foi de 19 (8-33) meses. A terapia combinada vs. iSGLT2 reduziu o risco de eventos por IC (HR 0,69; 95% IC 0,56-0.87) ou mortalidade por todas as causas (HR 0,68; IC 95% 0,54-0,86). A regressão multivariada de Cox, após pareamento com escore de propensão, confirmou o benefício da terapia combinada comparada ao iSGLT2 e ao AR GLP-1 em monoterapia. A terapia combinada de iSGLT2 e AR GLP-1 se associou à redução do risco de eventos de IC e mortalidade por todas as causas em comparação com a monoterapia nessa população.^87^

### 3.3 Cirurgia Bariátrica

#### 3.3.1. Obesidade Estágio 2 e Risco de DCVA Moderado/Alto ou Alto Risco de IC

**Table:** 

R24.	Grau de recomendação	Nível de evidência
**É RECOMENDADA** a indicação de cirurgia bariátrica para indivíduos com IMC ≥ 35 kg/m² e risco DASCV MODERADO/ALTO ou risco IC ALTO, quando o tratamento clínico disponível for insuficiente para promover perda de peso e melhora de fatores de risco CV de forma sustentada.	I	B

Sumário de evidências (R24):

- Uma revisão sistemática e metanálise de 18 estudos observacionais, baseados em banco de dados populacionais com mais de 1,5 milhão de pacientes, avaliou a incidência de doenças relacionadas à obesidade ou mortalidade global, após um seguimento mínimo de 18 meses, em pacientes submetidos à cirurgia bariátrica vs. grupo controle. A análise identificou que a cirurgia bariátrica está associada à redução da mortalidade por todas as causas (odds ratio [OR] 0,62; IC 95%: 0,55-0,69; p < 0,001) e da mortalidade CV (OR 0,50; IC 95%: 0,35-0,71; p < 0,001). Além disso, houve redução na incidência de diabetes tipo 2 (OR 0,39; IC 95%: 0,18-0,83), hipertensão (OR 0,36; IC 95%: 0,32-0,40) e dislipidemia (OR 0,33; IC 95%: 0,14-0,80).^88^
- Um estudo de coorte longitudinal avaliou 1.724 pacientes submetidos à cirurgia bariátrica (banda gástrica com derivação em Y-de-Roux), comparados com controles pareados para idade, IMC, sexo e escore de Framingham submetidos a tratamento clínico convencional e acompanhados por até 12 anos (mediana de 6.3 anos). Demonstrou-se que a cirurgia foi associada a uma redução de 42% no risco de MACE (HR 0,58; IC 95% 0,42-0,82; p = 0,0018), incluindo infarto do miocárdio, acidente vascular cerebral e IC congestiva. A redução observada na IC congestiva foi particularmente expressiva (HR 0,38; IC 95% 0,22-0,64; p = 0,0003). A melhora observada nos fatores de risco CV (colesterol total, HDL-colesterol, pressão arterial) ocorreu em até 1 ano, e em até 2 anos no escore de Framingham.^89^
- Em paralelo, um estudo observacional avaliou 20.235 pacientes com obesidade graus 2 ou 3 e diabetes tipo 2 no período de 2005 a 2010 nos EUA, onde 5.301 foram submetidos à cirurgia bariátrica e 14.934 serviram como controles, sendo pareados para idade, sexo, IMC e HbA1c. O desfecho primário foi a incidência de infarto agudo do miocárdio, angina instável, intervenção coronária percutânea ou cirurgia de revascularização miocárdica. Após 5 anos de seguimento, o grupo submetido à cirurgia bariátrica teve menor incidência do desfecho primário em relação ao grupo não-cirúrgico: 2,1% vs. 4,3% (HR 0,60, IC 95% 0,42-0,86), respectivamente. Houve também uma menor incidência de DAC no grupo cirúrgico em relação ao grupo controle não cirúrgico: 1,6% vs. 2,8% (HR 0,64, IC 95% 0,42-0,99).^90^
- O estudo observacional SOS demonstrou que a cirurgia bariátrica está associada à redução no risco de desenvolver IC em indivíduos com obesidade severa vs. pessoas com obesidade sob cuidado usual.^91^
- Nesses estudos, os efeitos da cirurgia bariátrica sobre a redução de eventos CV foram observados progressivamente após a normalização de parâmetros metabólicos, como pressão arterial, perfil lipídico e controle glicêmico. A redução de MACE não pode ser atribuída a um efeito direto ou imediato do procedimento cirúrgico sobre o sistema CV, já que a melhora dos fatores de risco CV ocorreu após perda de peso sustentada, e a comparação realizada com tratamento clínico de menor eficácia. Este painel considera que, na ausência de ECRs específicos, a indicação de cirurgia bariátrica na prevenção de eventos CV deverá ser realizada levando-se em conta o seu benefício em longo prazo na melhora de fatores de risco em pacientes com risco CV moderado ou alto e que não possuam acesso ou resposta clínica eficaz e sustentada às terapias antiobesidades atualmente disponíveis.

#### 3.3.2. Obesidade Estágio 2 e Insuficiência Cardíaca

**Table:** 

R25.	Grau de recomendação	Nível de evidência
**PODE SER CONSIDERADA** a indicação de cirurgia bariátrica em pacientes com IMC ≥35 kg/m² e IC estabelecida para promoção de perda de peso, melhora de fatores de risco e sintomas relacionados à IC, devendo ser avaliada com cautela e de acordo com o risco cirúrgico do paciente.	IIb	B

Sumário de evidências (R25):

- Embora a IC grave ou a disfunção sistólica possam ser consideradas contraindicações para a cirurgia bariátrica, alguns estudos indicam que a cirurgia pode ser considerada em pacientes com obesidade e com IC estável.
- Uma revisão sistemática com metanálise demonstrou que a cirurgia bariátrica está associada a uma redução nas hospitalizações por IC e benefícios na fração de ejeção do ventrículo esquerdo e na classe funcional da NYHA.^92^
- A cirurgia bariátrica demonstrou benefício na redução de fatores de risco CV e na melhoria da função cardíaca, incluindo a reversão do remodelamento cardíaco e melhorias na função sistólica e diastólica.^93^

**Table:** 

NOTA IMPORTANTE 7: Cirurgia Bariátrica em pacientes com IC
A segurança e a eficácia a longo prazo da cirurgia bariátrica em pacientes com IC ainda não foram totalmente estabelecidas, e mais estudos prospectivos são necessários para definir quais pacientes possam ser referidos com segurança para esse tipo de intervenção. A decisão de realizar cirurgia bariátrica em pacientes com IC deve ser individualizada, considerando o estado clínico do paciente, a presença de comorbidades e a capacidade de tolerar o procedimento cirúrgico. Uma abordagem multidisciplinar é recomendada para otimizar os resultados e minimizar os riscos associados.

**Table:** 

NOTA IMPORTANTE 8: Tratamento da Obesidade em Idosos
No contexto da população idosa, a conduta terapêutica deverá ser individualizada, já que há maior prevalência de obesidade sarcopênica, fragilidade, múltiplas comorbidades e polifarmácia. Todos estes fatores devem ser levados em consideração em especial no que se refere ao estabelecimento de metas terapêuticas de perda de peso. As recomendações desta diretriz se basearam nas melhores evidencias clínicas disponíveis (estudos ancilares e estudos clínicos randomizados controlados bem delineados), nos quais pessoas com idade avançada (acima de 75 anos) é subrepresentada. Na subanálise por subgrupos por faixas etárias em estudos de desfecho cardiovascular com AR GLP-1 AR, o beneficio cardiovascular foi observado de forma consistente entre todas as faixas etárias.^94^

**Table:** 

NOTA IMPORTANTE 9: Tratamento da Obesidade no contexto da saúde pública
Apesar da alta prevalência da obesidade no Brasil, ela permanece pouco contemplada em termos de políticas publicas no contexto das doenças crônicas não transmissíveis (DCNT) relacionadas às doenças cardiovasculares (DCV). A estratificação de risco CV proposta nesta diretriz fornece um racional para melhores decisões na escolha do tratamento antiobesidade. Reconhecendo que o risco cardiovascular nas pessoas com obesidade ocorre em um continuum, a abordagem baseada na classificação de risco CV pode auxiliar gestores formuladores de políticas publicas para a tomada de decisões na implementação de tratamentos para populações de maior risco CV.

## Conclusão

Considerando a crescente incidência da obesidade e sua associação bem estabelecida com a doença cardiovascular (DCV) e desfechos relacionados, a avaliação do risco cardiovascular deve ser um componente central no planejamento do tratamento da obesidade. Esta diretriz, desenvolvida em colaboração entre cinco das principais sociedades médicas brasileiras, aborda essa necessidade crítica ao fornecer estratégias baseadas em evidências para o tratamento da obesidade e a prevenção da DCV. Importante destacar que a diretriz considera o contexto específico de saúde pública da população brasileira, oferecendo recomendações que avaliam cuidadosamente os riscos e benefícios de cada abordagem terapêutica. Reconhecemos que a ampla implementação destas recomendações para a população brasileira representa um grande desafio. Como atualmente não há nenhum tratamento farmacológico antiobesidade disponível no Sistema Único de Saúde (SUS), entendemos que a priorização de intervenções com comprovada eficácia para redução de eventos cardiovasculares nos grupos de maior risco CV poderá auxiliar na tomada de decisões e planejamento de recursos por com foco no controle da obesidade e redução de suas complicações.

**Table:** 

RASTREAMENTO DE FATORES DE RISCO PARA DASCV E IC
**Hipertensão Arterial** Para o rastreamento e diagnóstico de hipertensão arterial em adultos, deve ser usado o critério de pressão sistólica ≥ 140 mmHg e/ou diastólica ≥ **90** mmHg em duas ou mais medições em diferentes ocasiões. Para pré-hipertensão, considerar pressão sistólica 130-139 mmHg e/ou diastólica **85-89** mmHg.
**Dislipidemia** O rastreamento universal para hipercolesterolemia é preconizado para crianças a partir dos 9 anos de idade e, se houver história de hipercolesterolemia na família, após os **2** anos de idade. O rastreamento deve ser realizado com medida de colesterol e triglicerídeos.
**Diabetes tipo 2** Todos os indivíduos com sobrepeso ou obesidade devem ser rastreados para diabetes tipo 2, com glicemia em jejum e hemoglobina glicada simultaneamente, a partir dos 35 anos. Antes dos 35 anos, o rastreamento é obrigatório se houver pelo menos um dos fatores de risco adicionais para diabetes (ver Quadro 1 ). O rastreamento também poderá ser considerado antes dos 35 anos, caso a caso, a critério do médico assistente e em comum acordo com o paciente.

**QUADRO 1:** FATORES DE RISCO QUE INDICAM RASTREAMENTO PARA DIABETES TIPO 2 EM PACIENTES COM OBESIDADE OU SOBREPESO

Glicemia de jejum prévia elevada (>100 mg/dL). Diabetes gestacional prévio. Mãe de recém-nascido grande para idade gestacional. História familiar de diabetes tipo 2 em parente de 1^o^grau. História de DCV clínica (SCA/IAM, DAC crônica, AVC isquêmico ou AIT, revascularização arterial), ou DCV subclínica (CAC >0 ou presença de placa em artéria carótida). Presença de hipertensão arterial, tratada ou não. HDL < 35 mg/dL. Triglicerídeos > 250 mg/dL. Síndrome de ovários policísticos. Presença de *acantose nigricans.* Sedentarismo. Doença hepática esteatótica metabólica (MASLD ou MASH). FINDRISC alto ou muito alto (Ver suplementos).
